# Function of AP2/ERF Transcription Factors Involved in the Regulation of Specialized Metabolism in *Ophiorrhiza pumila* Revealed by Transcriptomics and Metabolomics

**DOI:** 10.3389/fpls.2016.01861

**Published:** 2016-12-09

**Authors:** Nirin Udomsom, Amit Rai, Hideyuki Suzuki, Jun Okuyama, Ryosuke Imai, Tetsuya Mori, Ryo Nakabayashi, Kazuki Saito, Mami Yamazaki

**Affiliations:** ^1^Department of Molecular Biology and Biotechnology, Graduate School of Pharmaceutical Sciences, Chiba UniversityChiba, Japan; ^2^Department of Research and Development, Kazusa DNA Research InstituteChiba, Japan; ^3^RIKEN Center for Sustainable Resource ScienceKanagawa, Japan

**Keywords:** camptothecin, AP/ERF, *Ophiorrhiza pumila*, RNA-seq, secondary metabolism, metabolome

## Abstract

The hairy roots (HR) of *Ophiorrhiza pumila* produce camptothecin (CPT), a monoterpenoid indole alkaloid used as a precursor in the synthesis of chemotherapeutic drugs. *O. pumila* HR culture is considered as a promising alternative source of CPT, however, the knowledge about the biosynthetic pathway and regulatory mechanism is still limited. In this study, five genes that encode AP2/ERF transcription factors, namely *OpERF1*–*OpERF5*, were isolated from HR of *O. pumila*. Phylogenetic analysis of AP2/ERF protein sequences suggested the close evolutionary relationship of *Op*ERF1 with stress-responsive ERF factors in Arabidopsis and of *Op*ERF2 with ERF factors reported to regulate alkaloid production, such as ORCA3 in *Catharanthus roseus, NIC2* locus ERF in tobacco, and JRE4 in tomato. We generated the transgenic HR lines of *O. pumila*, ERF1i and ERF2i, in which the expression of *OpERF1* and *OpERF2*, respectively, was suppressed using RNA interference technique. The transcriptome and metabolome of these suppressed HR were analyzed for functional characterization of *Op*ERF1 and *Op*ERF2. Although significant changes were not observed in the metabolome, including CPT and related compounds, the suppression of *OpERF2* resulted in reduced expression of genes in the 2-*C*-methyl-d-erythritol 4-phosphate and secologanin-strictosidine pathways, which supply a precursor, strictosidine, for CPT biosynthesis. Furthermore, while it was not conclusive for *Op*ERF1, enrichment analysis of differentially expressed genes in the suppressed HR showed that the gene ontology terms for oxidation-reduction, presumably involved in secondary metabolite pathways, were enriched in the ERF2i downregulated gene set. These results suggest a positive role of *Op*ERF2 in regulating specialized metabolism in *O. pumila*.

## Introduction

Plants produce a broad array of secondary metabolites that serve special functions, such as protection from biotic and abiotic threats, attracting insects and animals for pollination and seed dispersal among others. A number of these phytochemicals have been widely used by humans for agricultural, medical, nutritional, and industrial purposes (Croteau et al., 2000; Krishna, 2003; Bakkali et al., 2008; Mithöfer and Boland, 2012; Jacobo-Velázquez et al., 2015). One of the commercially important classes of plant secondary metabolite is alkaloid, which comprises of organic compounds containing nitrogen atom in diverse structures. Different types of alkaloids exhibit a wide range of pharmaceutical activities that are beneficial for clinical treatments, for example, the antispasmodic activity of the tropane alkaloid hyoscyamine, antimalarial activity of the quinoline alkaloid quinine, and antitumor activity of the monoterpenoid indole alkaloids (MIA) vincristine and vinblastine (Amirkia and Heinrich, 2014).

Camptothecin (CPT) was first isolated from *Camptotheca acuminata* (Wall et al., 1966), and it is currently well recognized as an antitumor agent because of its unique mechanism of action through the inhibition of DNA topoisomerase I (Hsiang et al., 1985). Although, CPT belongs to the structural family of quinoline alkaloids, from the biosynthetic view point CPT constitutes a MIA, which is one of the largest groups of bioactive plant alkaloids. Interestingly, CPT is produced by several plant species that are taxonomically distant from *C. acuminata*, such as *Nothapodytes foetida* (Govindachari and Viswanathan, 1972) and *Ophiorrhiza pumila* (Aimi et al., 1989). Semi-synthetic CPT derivatives such as topotecan and irinotecan, in which low water solubility and potent toxicity of CPT were modified, were approved for the treatment of recurrent small-cell lung cancer (Garst, 2007), ovarian cancer (Coleman, 2002), and colorectal cancer (Cunningham et al., 2001). Furthermore, other CPT derivatives such as cositecan (BNP1350) (Munster and Daud, 2011) and belotecan (CKD-602) (Kim et al., 2010), are currently undergoing clinical trials. Growing demand for these drugs requires the increasing amount of naturally-derived CPT as a lead compound for synthesis. Global CPT supply from extracts of bark and seeds of *C. acuminata* and *N. foetida* (Lorence and Nessler, 2004) has raised the concern of environmental sustainability and CPT supply chain sufficiency and thus led to the necessity of the establishment of alternative sources (Sadre et al., 2016). Several studies have attempted to develop cell culture systems for CPT-producing plants but only a few could achieve high yield of CPT. For example, callus and hairy roots (HR) cultures of *C. acuminata* produced 0.1 and 0.236% dry weight of CPT, respectively (Wiedenfeld et al., 1997; Lorence et al., 2004).

HR cultures of *O. pumila* accumulated 0.1% dry weight of CPT, which was at least 2.5 times higher than the level accumulated in the leaves (Saito et al., 2001). Considering a short proliferation period and successful establishment of tissue culture in an upscale bioreactor system (Sudo et al., 2004), *O. pumila* HR is a potential source of *in vitro* CPT production, and it serves as a model for studying CPT biosynthesis. To date, biosynthetic genes that encode catalytic enzymes, tryptophan decarboxylase (TDC), geraniol 10-hydroxylase (G10H), secologanin synthase (SLS), and strictosidine synthase (STR), were isolated and characterized in *O. pumila* (Yamazaki et al., 2003a,b). The incorporation of labeled [1-^13^C] glucose into HR of *O. pumila* indicated that the secologanin moiety of the CPT molecule is produced via 2-*C*-methyl-d-erythritol 4-phosphate (MEP) pathway and tryptamine is from the shikimate pathway (Yamazaki et al., 2004). The biosynthetic intermediates were searched using non-targeted metabolite profiling of the HR with the suppression of *TDC* and *SLS* (Asano et al., 2013). Using deep transcriptome analysis and untargeted metabolic profiling of *O. pumila* HR, the candidate genes in the specialized metabolism were profiled in comparison with those in the cell suspension culture (CSC) that doesn't produce CPT (Yamazaki et al., 2013). Recently, Rohani et al. (2016) reported the characterization of a suppressor, *Op*MYB1, involved in the regulation of specialized metabolism in *O. pumila*. Their study demonstrated that overexpression of *OpMYB1* in HR negatively affected the transcript level of *TDC* and resulted in reduced accumulation of CPT. Furthermore, some other transcription factor genes were profiled as genes specifically expressed in HR. The HR culture of *O. pumila* is a robust system for studying CPT biosynthesis in plants. To utilize HR effectively as an alternative source for CPT, the ability to control and manipulate the system is indispensable. Information on the production pathway, including enzymes, intermediates and regulatory factors that influence CPT and other related metabolites production, is necessary.

The regulatory mechanism underlying MIA biosynthesis has been intensively studied in *Catharanthus roseus*. The jasmonate signaling pathway regulates the production of vinca alkaloids. Transcription factors *Cr*MYC2, *Cr*ORCA2, *Cr*ORCA3, and *Cr*WRKY1 were identified to be involved in this signal transduction (Menke et al., 1999; van der Fits and Memelink, 2000, 2001; Suttipanta et al., 2011; Zhang et al., 2011; Schluttenhofer et al., 2014). Among them, *Cr*ORCA2 and *Cr*ORCA3 are classified as APETHALA2/Ethylene Response Factor (AP2/ERF). AP2/ERF represents a superfamily of transcription factor that plays an important role in plant biological systems, such as growth and development (Chuck et al., 1998; Ohto et al., 2009), response to stimuli (Licausi et al., 2010; Mizoi et al., 2012; Zhao et al., 2012), and biosynthesis of primary and secondary metabolites (van der Fits and Memelink, 2000, 2001; Zhang et al., 2005; Shoji et al., 2010; Todd et al., 2010; Cárdenas et al., 2016; Thagun et al., 2016). On the basis of the number of conserved AP2/ERF domain and the existence of a family-specific B3 domain, AP2/ERFs are further classified into three families: AP2, ERF, and RAV. Among these families, the ERF family is the largest and is characterized by a single AP2/ERF domain. In 2006, Nakano et al. subcategorized the Arabidopsis ERF family into 12 groups on the basis of phylogenetic relationships, exon-introns, and protein motifs (Nakano et al., 2006). In their study, group I to IV correspond to dehydration-responsive element binding-protein (DREB) factors, and group V–X, group VI-L, and group Xb-L correspond to ERF factors. This classification provides a perspective on the biological functions of each group, as demonstrated by several members of the group IX ERF from different plants that are involved in regulation of secondary metabolite biosynthesis. For example, *Cr*ORCA2 and *Cr*ORCA3 from *C. roseus* positively regulate the expression of genes in vinca alkaloid biosynthesis (Menke et al., 1999; van der Fits and Memelink, 2000, 2001). *Nb*ERF1 from *Nicotiana benthamiana* caused a reduction in nicotine alkaloids accumulation when the expression was suppressed (Todd et al., 2010). A cluster of *ERFs* in the *NIC2* locus of *Nicotiana tabacum* control nicotine biosynthesis in tobacco (Shoji et al., 2010). Recently, the orthologs of GAME9/JRE4 were reported to modulate steroidal glycoalkaloids (SGA) in tomato and potato (Cárdenas et al., 2016; Thagun et al., 2016). *Aa*ERF1 and *Aa*ERF2 from *Artemisia annua* L. positively regulate artemisinin biosynthesis (Yu et al., 2012).

In this study, ERF family genes, *OpERF1, OpERF2, OpERF3, OpERF4*, and *OpERF5*, were isolated from CPT-producing HR of *O. pumila*. Their expression profiles and evolutional relationships with reported secondary metabolite-regulating AP2/ERFs and Arabidopsis AP2/ERFs were analyzed. For further characterization, RNA interference (RNAi) transgenic HR with suppressed *OpERF1* (ERF1i) or *OpERF2* (ERF2i) were generated. Using the transcriptome and metabolome approach, a positive role of *Op*ERF2 in regulating the MEP and secologanin-strictosidine pathways, which produce a precursor for CPT biosynthesis, and the possible involvement of *Op*ERF1 in stress response were revealed.

## Materials and methods

### Plant materials

*O. pumila* transgenic HR were maintained in the condition as described by Saito et al. (2001). Collected HR were immediately frozen in liquid nitrogen and homogenized using multibeads shocker (Yasui Kikai, Japan).

### RNA extraction and cDNA synthesis

Total RNA was extracted from homogenized 3-week-old HR using RNeasy Plant Mini Kit (Qiagen, Germany) and treated with RNase-free DNase (Qiagen, Germany). First strand cDNA synthesis was carried out using Superscript® VILO™ Reverse Transcriptase (Invitrogen, USA) according to the manufacturer's instructions.

### Isolation and cloning of *OpERFs*

Isolation of differentially expressed genes between HR and CSC using PCR-select™ cDNA subtraction (Clontech, Japan) was performed according to previously described by Bunsupa et al. (2011). HR-specific AP2/ERF unigenes were selected from *de novo* transcriptome assembly (Yamazaki et al., 2013) using log_2_ reads per kilobase of transcript per million mapped reads (RPKM) of HR/CSC ≥1 as a cut-off value. Full-length coding sequences of *OpERF1* (LC183910), *OpERF2* (LC171328), *OpERF3* (LC171329), *OpERF4* (LC183911), and *OpERF5* (LC183912) were obtained from 3′ to 5′ RACE using SMART™ RACE cDNA amplification kit (Clontech, Japan) according to the manufacturer's instructions. Amplified RACE fragments were subjected for DNA sequencing by Eurofins Genomics, Japan.

### Phylogenetic analysis

Full-length amino acid sequences of AP2/ERFs were aligned by ClustalW program (Larkin et al., 2007) in BioEdit software version 7.2.5 (Hall, 1999). Phylogenetic tree was constructed using MEGA6 software (Tamura et al., 2013) with neighbor joining algorithm and 1000 bootstrap replicates. Sequence data of alkaloid-regulating AP2/ERFs and terpenoid-regulating AP2/ERFs used in the analysis can be found under the following accession numbers: *Aa*ERF1 (AEQ93554.1), *Aa*ERF2 (AEQ93555.1), *Aa*ORA (AGB07586.1), *Cr*ORCA2 (CAB93940.1), *Cr*ORCA3 (ABW77571.1), *Nb*ERF1 (ADH04266.1), *Nt*ERF32 (NP_001311965.1), *Nt*ERF189 (NP_001312507.1), *Nt*ORC1 (XP_016478305.1), and *Sl*JRE4 (Solyc01g090340). Arabidopsis ERF family sequences were retrieved from Plant Transcription Factor Database 3.0 (http://planttfdb.cbi.pku.edu.cn/; Jin et al., 2014).

### Binary vector construction and induction of RNAi hairy roots

RNAi target sequences of 800 base pair (bp) from *OpERF1* and 230 bp from *OpERF2* (Figure S1) were amplified from cDNA of *O. pumila* HR. The Gateway® cloning technology (Invitrogen, USA) was utilized in a recombination reaction to insert target sequences into pGWB80, a binary vector system for RNAi used previously in *O. pumila* (Figure S1; Asano et al., 2013). The gene-specific *att*B primers used in amplification of target sequences are as follows: OpERF1i-*att*B1 (5′-wordAAAAAGCAGGCTACCTGATTATTTTTATTACGAAACCCCCG-3′), OpERF1i-*att*B2 (5′-wordAGAAAGCTGGGTACGACTCATAAGCCATGAGCTCCT-3′), OpERF2i-*att*B1 (5′-wordAAAAAGCAGGCTTC-TCTCTGATCTTGCCATCCTTG-3′), and OpERF2i-*att*B2 (5′-wordGAAAGCTGGGTT-CGTCAATGACACGTTCTTTCC-3′). The coding region of *Escherichia coli* β*-glucuronidase* (*GUS)* was used as a target sequence in the negative control construct. Fidelity of inserted RNAi target sequences was confirmed by DNA sequencing (Eurofins Genomics, Japan). Transformation of binary vectors into *Agrobacterium rhizogenes* (pRi15834) and hairy roots induction were operated according to Asano et al. (2013). Three hundred milligrams per liter cefotaxime was used in *Agrobacterium* disinfection and a combination of 25 mg/L hygromycin and 25 mg/L kanamycin was used in antibiotic selection.

### Quantitative real-time PCR (qRT-PCR)

cDNA synthesized from total RNA was used as an amplification template in qRT-PCR. PCR reaction was performed using Power SYBR® Green PCR Master Mix (Thermo Fisher Scientific, USA) on Applied Biosystems StepOnePlus™ Real-Time PCR System (Thermo Fisher Scientific, USA). Gene specific primers used in qRT-PCR are listed in Table S1. A housekeeping gene β-*tubulin* was used for normalization. Relative expression fold change of the target genes in ERF1i and ERF2i vs. GUSi was calculated by comparative C_T_ method. The significant differences compared to average of GUSi were determined by *t*-test (*p* < 0.05).

### RNA sequencing (RNA-seq) and raw data processing

Total RNA extracted from each transgenic lines were subjected for cDNA library preparation and sequencing at Kazusa DNA Research Institute (Chiba, Japan) on Illumina HiSeq™ 2000 sequencer (Illumina Inc., USA). Raw read pre-processing, alignment of clean reads to a reference *O. pumila de novo* assembly (Rohani et al., 2016), transcript abundant estimation and expression level in fragment per kilobase exon per million mapped fragment (FPKM) were performed as described by Rai et al. (2016a). Raw read sequences of six RNAi HR lines were deposited in NCBI's Gene Expression Omnibus (Accession number GSE89674; Edgar et al., 2002).

### Differential gene expression analysis and gene ontology (GO) enrichment analysis

Expression fold change of genes in ERF1i and ERF2i relative to GUSi was calculated using DESeq2 (Love et al., 2014) by assigning log_2_ FPKM ≥ 1 and false discovery rate < 0.05 as a threshold. Upregulated and downregulated gene sets were used in GO analysis based on Fisher's exact test with a *p*-value cut-off 0.05, using *O. pumila de novo* assembly as a reference set as described by Rai et al. (2016b). Blast2GO version 3.0 (Biobam, Spain) was used for the assignment of GO term and visualization of the GO functional classification. Only GO categories passing the statistical criteria were chosen for further discussion.

*O. pumila de novo* transcriptome assemblies were subjected to tBLASTx alignment against transcripts from the MEP and secologanin-strictosidine pathways from *C. roseus* (Van Moerkercke et al., 2013; Miettinen et al., 2014) and the jasmonic acid biosynthesis pathway from Arabidopsis to identify their corresponding contigs in *O. pumila* (Table S2). Top hit contigs of each enzyme with *p* < 0.05, positive alignment ≥70%, and length >500 bp, were used in functional analysis.

### Untargeted metabolite analysis using LC-QTOF-MS

The homogenized HR were freeze dried using FDU-2200 freeze dryer (Tokyo Rikakikai, Japan) and metabolites were extracted with 50 μL of 80% MeOH containing 2.5 μM lidocaine and 10-camphor sulfonic acid per milligram dry weight using a mixer mill with zirconia beads for 7 min at 18 Hz at 4°C. After centrifugation for 10 min, the supernatants were filtered using an HLB μElution plate (Waters). The extracts were analyzed using LC-QTOF-MS according to the condition described by Nakabayashi et al. (2014) with modifications as following; LC: gradient program, 0.5%B at 0 min, 5%B at 0.1 min, 80%B at 10 min, 99.5%B at 10.1 min, 99.5%B at 12.0 min, 0.5%B at 12.1 min, and 0.5%B at 15.0 min; flow rate, 0.3 ml/min at 0 min, 0.3 ml/min at 10 min, 0.4 ml/min at 10.1 min, 0.4 ml/min at 14.4 min, and 0.3 ml/min at 14.5 min; MS detection: mass range, *m/z* 50–1500; polarity, positive. MS/MS data was acquired in the ramp mode as the following analytical conditions: (1) MS: mass range, *m/z* 50–1500; scan duration, 0.1 s; inter-scan delay, 0.014 s; data acquisition, centroid mode; polarity, positive; and (2) MS/MS: mass range, *m/z* 50–1500; scan duration, 0.02 s; inter-scan delay, 0.014 s; data acquisition, centroid mode; polarity, positive collision energy, ramped from 10 to 50 V. In this mode, MS/MS spectra of the top 10 ions (>1000 counts) in an MS scan were automatically obtained. If the ion intensity was <1000, MS/MS data acquisition was not performed and moved to next top 10 ions. Data acquisition was performed using Progenesis CoMet (Nonlinear Dynamics, Durham, NC, USA). Peaks with intensity less than 2000 were treated as noise and not proceeded for MS/MS data acquisition. Peak normalization was conducted by comparison with internal standard (lidocaine). Chemical assignment was conducted using *m/z* values reported previously as a reference (Yamazaki et al., 2013) with tolerance 0.01 Da.

## Results and discussion

### Isolation of hairy roots specific AP2/ERFs

A previous study reported higher accumulation of CPT and putative intermediates in the HR culture of *O. pumila*, whereas no synthesis was observed in the CSC derived from HR (Saito et al., 2001). This opposite secondary metabolite profile for CPT and related intermediates implies that genes associated with the production of these compounds are active in HR and limited/not active in CSC. Therefore, using comparative expression analysis, we identified AP2/ERFs that are specially expressed in HR when compared with CSC. From the data derived by PCR-select cDNA subtraction (Rohani et al., 2016), six out of 353 HR-specific fragments were annotated as ERF-like proteins. Full-length cDNA cloning revealed that all these six fragments constitute a part of 903-bp cDNA sequence (designated as *OpERF1*). Next we evaluated a more comprehensive data set acquired by deep transcriptome analysis (Yamazaki et al., 2013) and identified 95 AP2/ERF annotated unigenes, with 31 unigenes being HR-specific (Table S3). As expected, among these 31 HR-specific AP2/ERFs, a homolog of *OpERF1*, unigene27166_All was also included. We further reviewed the annotation list of these unigenes and could narrow down four unigenes with a high sequence similarity with the functionally characterized AP2/ERFs (Table S3). Unigene18253_All (designated as *OpERF2*) showed 68% sequence identity to *Cr*ORCA3, an AP2/ERF transcription factor of *C. roseus* that regulates the expression of key genes in TIA biosynthesis (van der Fits and Memelink, 2000, 2001), and 65% sequence identity to *Nb*ERF1, which is involved in nicotine alkaloids biosynthesis in *N. benthamiana* (Todd et al., 2010). Unigene26293_All (designated as *OpERF3*) showed 69% identity to Arabidopsis *At*ERF13, a member of the group IX ERF as *Cr*ORCA2 and *Cr*ORCA3. Unigene34283_All (designated as *OpERF4*) and Unigene37445_All (designated as *OpERF5*) showed 60 and 88% identity, respectively, to pathogen response-related *Nt*ERF5 (Fischer and Dröge-Laser, 2004). These annotations formed the basis for selection of these four unigene candidates for further characterization. Cloning for their full-length coding sequences was performed using 3′ and 5′ RACE and top hit results from BLAST search of full-length *Op*ERF1 to *Op*ERF5 against NCBI's protein database are listed in Table 1.

**Table 1:** **Top hit from BLAST search of full-length *Op*ERF1 to *Op*ERF5 against NCBI's protein database**.

**Gene**	**Unigene**	**Annotation**	**Accession number**
*OpERF1*	Unigene27166_All	PREDICTED: ethylene-responsive transcription factor ERF071 isoform X1 [*Nicotiana tomentosiformis*]	XP_009608570.1
*OpERF2*	Unigene18425_All	PREDICTED: ethylene-responsive transcription factor 13-like [*Nicotiana tomentosiformis*]	XP_009593489.1
*OpERF3*	Unigene26293_All	Ethylene-responsive transcription factor ERF053 family [*Cajanus cajan*]	KYP46887.1
*OpERF4*	Unigene34283_All	unnamed protein product [*Coffea canephora*]	CDP01664.1
*OpERF5*	Unigene37445_All	PREDICTED: ethylene-responsive transcription factor 1B-like [*Nicotiana sylvestris*]	XP_009764685.1

### AP2/ERF domain and phylogenetic analysis of *Op*ERFs

**Figure 1:**
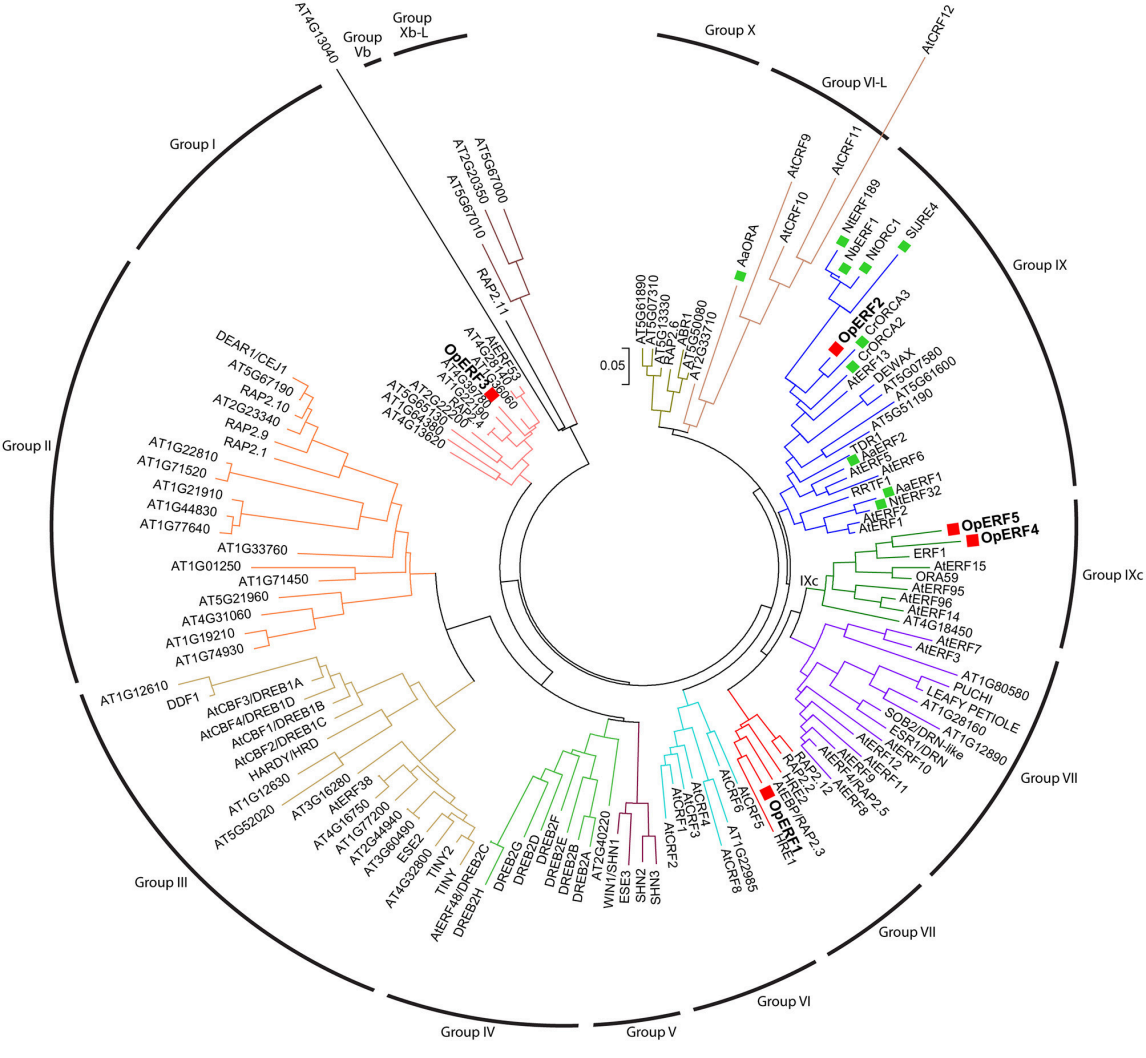
**Phylogenetic tree of *Op*ERF1 to *Op*ERF5, alkaloid-regulating AP2/ERFs, terpenoid-regulating AP2/ERFs, and Arabidopsis AP2/ERFs**. *Op*ERF1 to *Op*ERF5 are marked with a red squares. Alkaloid- and terpenoid-regulating AP2/ERFs are marked with a green squares. The tree was generated based on the alignment of full-length amino acid sequences using neighbor-joining algorithm by MEGA6 software. The scale bar shows the evolutionary distances. ERF group numbers based on the classification by Nakano et al. (2006) are shown. Op, *Ophiorrhiza pumila*; Aa, *Artemisia annua*; At, *Arabidopsis thaliana*; Cr, *Catharantus roseus;* Nt, *Nicotiana tabacum*; Nb, *Nicotiana benthamiana*; Sl, *Solanum lycopersicum*. The genes which species name are not mentioned are from Arabidopsis (van der Fits and Memelink, 2000, 2001; Shoji et al., 2010; Todd et al., 2010; Yu et al., 2012; Lu et al., 2013; Sears et al., 2013; Cárdenas et al., 2016; Thagun et al., 2016).

**Figure 2:**
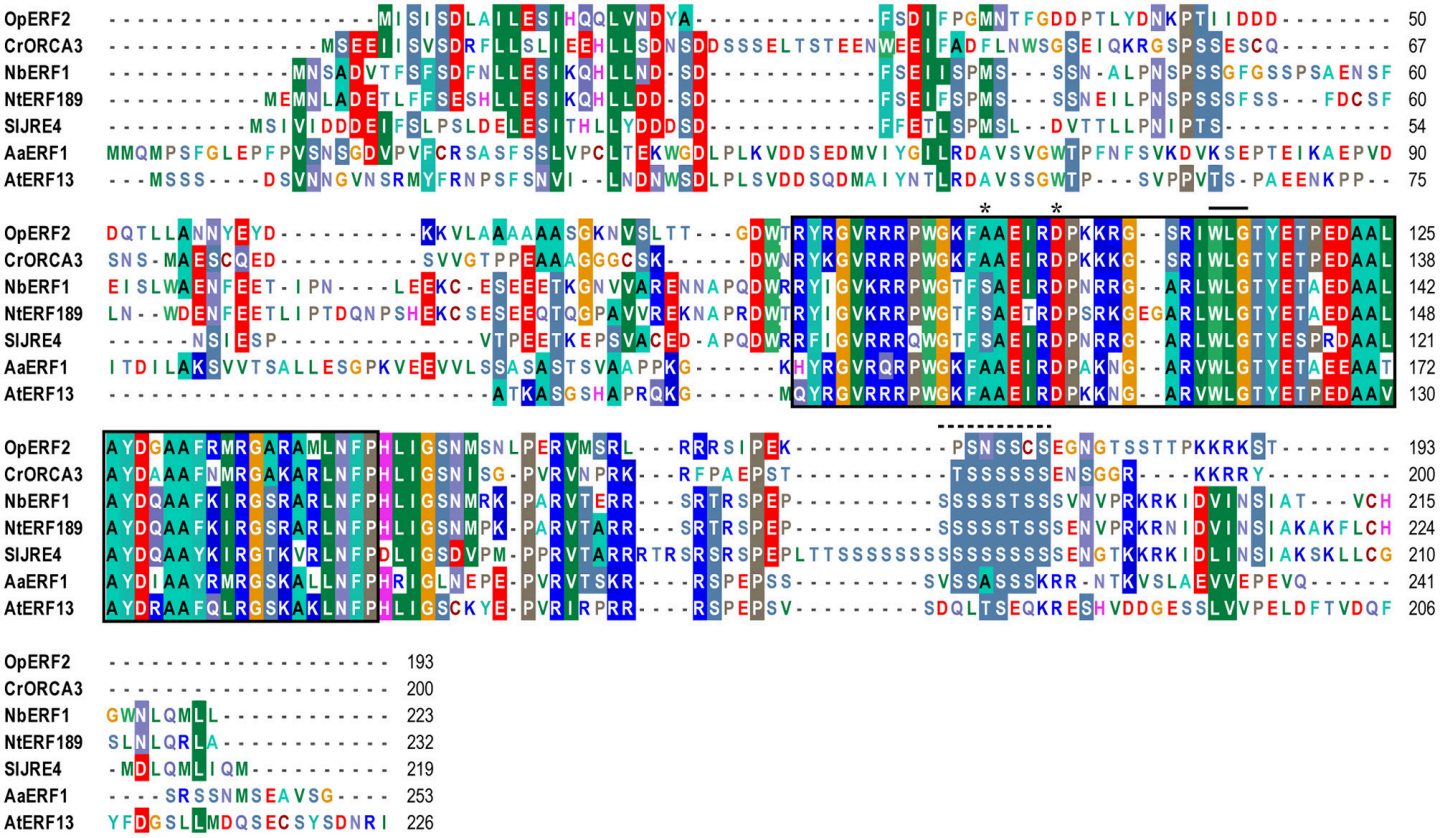
**Amino acid sequence alignment of *Op*ERF2 and secondary metabolite-regulating AP2/ERFs and Arabidopsis AP2/ERF**. Op, *Ophiorrhiza pumila*; At, *Arabidopsis thaliana*; Cr, *Catharantus roseus;* Nt, *Nicotiana tabacum*; Nb, *Nicotiana benthamiana*; Sl, *Solanum lycopersicum*. The sequences were aligned using ClustalW in Bioedit software version 7.2.5. AP2/ERF domain is enclosed in a black box. Asterisks indicate the reserved amino acids at position 14 and 19 of AP2/ERF domain. Solid bar indicates WLG motif. Dashed line indicates serine-rich region.

A phylogenetic tree was constructed using the amino acid sequences of all five *Op*ERFs identified in this study, together with known alkaloid-regulating AP2/ERFs, terpenoid-regulating AP2/ERFs, and Arabidopsis AP2/ERFs, to portray their evolutionary relationships (Figure 1). *Op*ERF1 was clustered in the group VII ERF clade. Several members of the group VII ERF in Arabidopsis have been reported to play an important role in biotic and abiotic stress responses. For example, hypoxia responsive ERF (HRE) 1 and HRE2 respond to a low oxygen environment (Licausi et al., 2010), and RAP2.2 plays a role in *Botrytis cinerea* resistance and ethylene response (Zhao et al., 2012). *Op*ERF2 was assigned to the clade that is composed of group IX ERFs, with the members previously reported to be involved in specialized metabolism. Interestingly, *Op*ERF2 exhibited a closer relationship with *C. roseus* ORCA2 and ORCA3 that regulate MIA biosynthesis than with *Sl*JRE4, *Nb*ERF1, *Nt*ERF189, and *Nt*ORC1, the ERFs from *Solanaceae* family that regulate SGA and nicotine alkaloids biosynthesis (van der Fits and Memelink, 2000, 2001; Shoji et al., 2010; Todd et al., 2010; Cárdenas et al., 2016; Thagun et al., 2016) or *Aa*ERF1 and *Aa*ERF2 from *Asteraceae* family that regulate terpenoid biosynthesis (Yu et al., 2012). *Op*ERF4 and *Op*ERF5 were classified into a separated cluster that exclusively consists of Arabidopsis group IXc ERFs. Several members of this group, such as ERF1 (AT3G23240.1), *At*ERF14, and *At*ERF15 act as strong transcription activators in defense response (Oñate-Sánchez and Singh, 2002; Zhang et al., 2015). *Op*ERF3, interestingly, was designated to the group I ERF clade composed of DREB members. Two functionally characterized members of this group, RAP2.4 and *At*ERF53, are related to abiotic stress responses (Lin et al., 2008; Hsieh et al., 2013). Alignment of full-length deduced amino acid sequences of the *Op*ERF1 to *Op*ERF5 showed the ERF family-specific WLG motif in all *Op*ERF sequences. Two characteristic amino acids among ERF members, alanine at position 14 and aspartic acid at position 19, were observed in the AP2/ERF domain of *Op*ERF1, *Op*ERF2, *Op*ERF4, and *Op*ERF5. On the other hand, valine and leucine, the characteristic amino acids among DREB members, were found at the respective positions in *Op*ERF3 (Liu et al., 1998; Sakuma et al., 2002; Nakano et al., 2006; Figure S2A). This difference in the AP2/ERF domains within AP2/ERFs suggests that *Op*ERF3 might have a distinct function. In the alignment of *Op*ERF2, secondary metabolite-regulating ERF sequences and Arabidopsis ERF sequence, the serine substitutes at position 14 of AP2/ERF domain were found in *Solanaceae* ERFs. A serine-rich regions, which has been proposed to be involved in transactivation of AP2/ERF transcription factor (Riechmann and Meyerowitz, 1998), exist in C-terminal region of all sequences (Figure 2). The sequence alignment of the group VII ERFs clearly showed a characteristic N-terminal MCGGAI(I/L) motif (Tournier et al., 2003) in all sequences (Figure S2B). Therefore, *Op*ERF1 may possess a similar function as the members of this group.

Taking into account that *OpERF1* is dominantly expressed (RPKM 21.229) among the five isolated *OpERFs* (Table S3) with a potential role in biotic and abiotic stress responses and *OpERF2* is the second highly expressed (RPKM 6.433; Table S3) with a close phylogenetic relationship with secondary metabolite-regulating AP2/ERFs, we selected these two AP2/ERFs for further functional characterization.

### Generation of RNAi transgenic hairy roots

**Figure 3:**
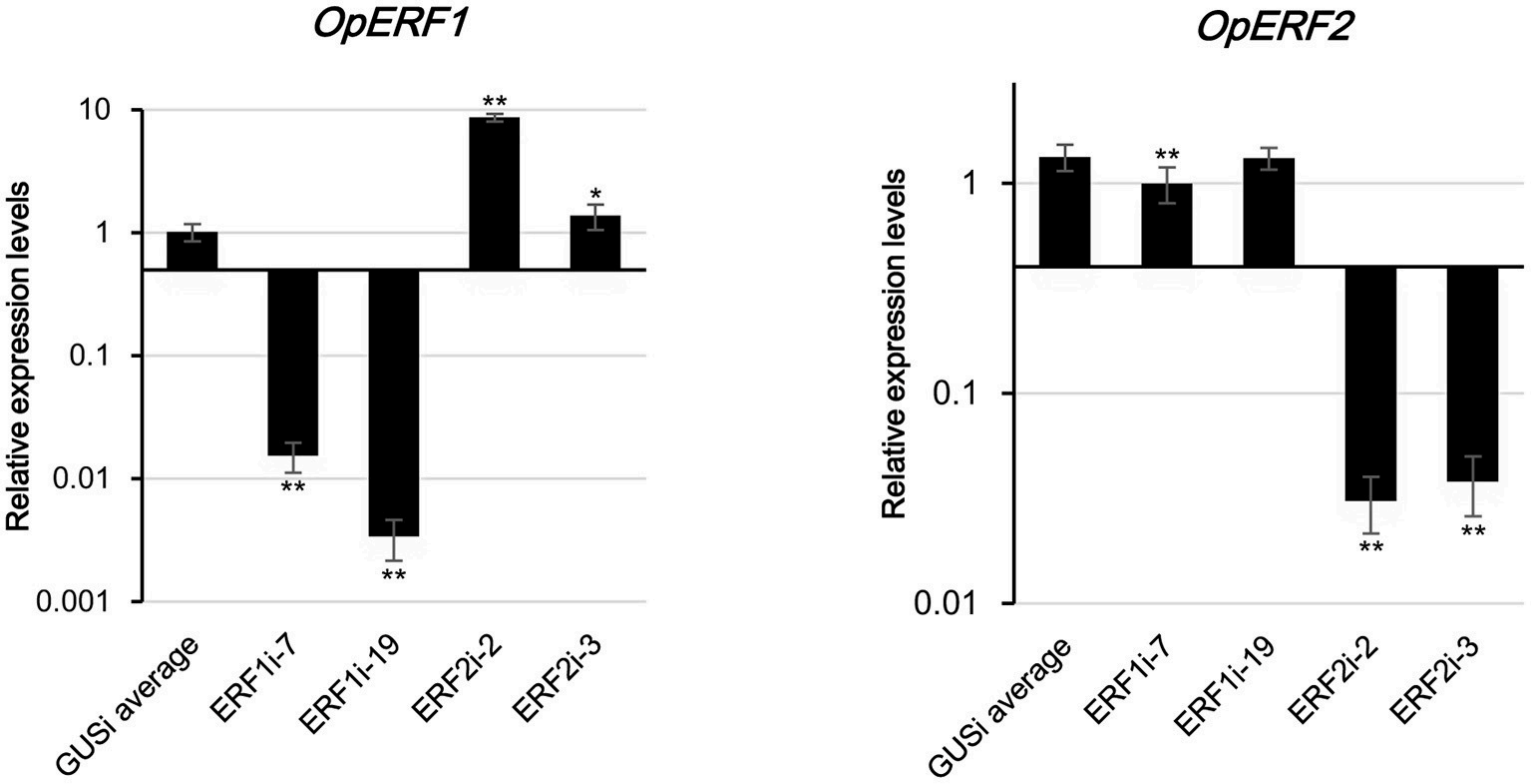
**Expression levels of *OpERF1* and *OpERF2* in selected RNAi lines**. Data represents means of 4 biological replicates ± standard deviation and experiments were repeated twice. Asterisks indicate significant differences compared to average of GUSi (*t*-test, ^*^p < 0.05, ^**^p < 0.01).

Transgenic research has been performed by the scientists for the functional characterization of genes. By perturbing the expression of the target transcript by using gain and loss of function coupled with multiomics analysis, biological importance, and functionality of genes of interest could be understood (Reuben et al., 2013; Verma et al., 2015). For functional characterization, transgenic HR lines with suppressed expression of *OpERF1* (ERF1i) and *OpERF2* (ERF2i) were generated using RNAi technique, and the β*-glucuronidase* gene was used in the negative control lines (GUSi). Expression levels of target genes in each transformation event were investigated using qRT-PCR (Figure S3). We obtained transgenic lines with strong suppression of target genes, with <0.02-fold expression of *OpERF1* in ERF1i and <0.04-fold expression of *OpERF2* in ERF2i when compared with the expression levels in GUSi. Two of the transgenic HR lines, each with the lowest expressions of *OpERF1* (ERF1i-7 and ERF1i-19) and *OpERF2* (ERF2i-2 and ERF2i-3), and two negative control GUSi lines (GUSi-9 and GUSi-10) were subjected to RNA-seq and untargeted metabolome analysis (Figure 3).

### RNA-seq based transcriptome profiling of transgenic HR lines

RNA-seq performed for the six transgenic HR lines generated more than 13 million paired end raw reads in each library, which provided more than 88 million paired end raw reads in total. Removal of adapter sequences, low-quality reads, and reads shorter than 50 bp yielded a total of over 84 million high-quality paired end clean reads (Table 2). Clean reads from individual libraries were aligned with previously described and annotated *O. pumila de novo* transcriptome assembly (Rohani et al., 2016) using Bowtie 2.0 (Langmead et al., 2009), and transcript abundance in FPKM was estimated using the RSEM program (Li and Dewey, 2011). More than 88% of the clean reads from each library could be aligned to a reference *de novo* assembly. The full-length coding sequences of *OpERF1* and *OpERF2* cloned in this study can be aligned with assembled contigs c15284_g1_i1 and c17706_g2_i1, respectively, with a high percentage of sequence identity (more than 98%) and low *E*-value.

**Table 2:** **Number of reads and aligned sequences from RNA-seq of *O. pumila* transgenic hairy roots**.

**Library**	**Raw read pairs**	**Paired end clean reads**	**Unpaired reads (F and R)**	**% Clean reads aligned to transcriptome assembly**
GUSi-9	15,205,047	14,607,832 (96.07%)	485,889 (3.58%)	88.9
GUSi-10	15,924,982	14,991,951 (94.14%)	868,098 (5.45%)	88.58
ERF1i-7	15,298,117	14,653,295 (95.78%)	589,133 (3.85%)	88.39
ERF1i-19	14,670,943	13,995,511 (95.40%)	620,187 (4.23%)	88.32
ERF2i-2	13,849,296	13,289,005 (95.95%)	512,326 (3.7%)	88.62
ERF2i-3	13,690,821	13,118,609 (95.82%)	523,473 (3.82%)	88.29

F, forward; R, reverse.

**Figure 4:**
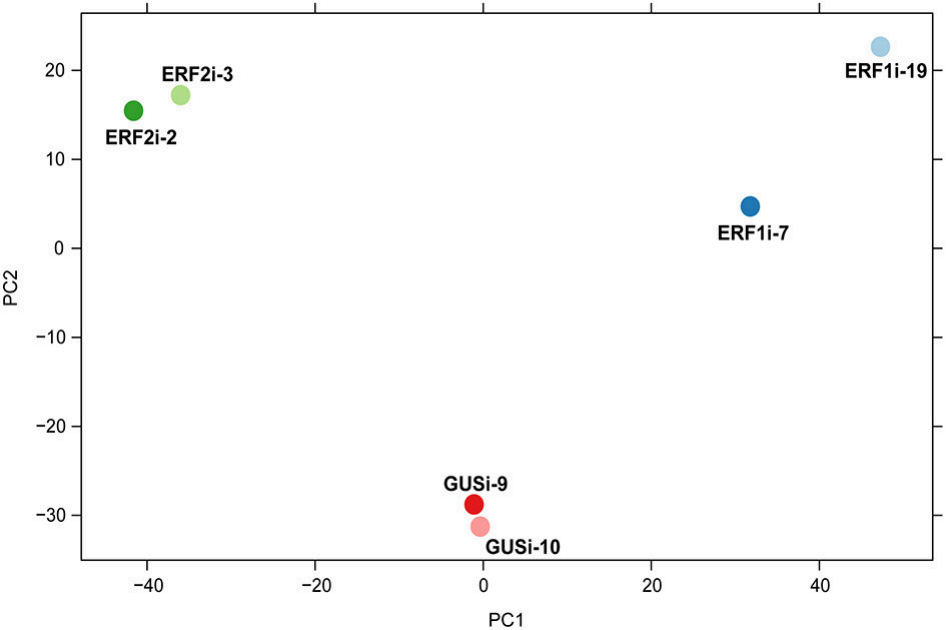
**PCA score plot generated from a transcriptome data of RNAi lines**.

Principal component analysis (PCA), a powerful multivariate data analysis approach, assists visualization of the relationship between samples with high-dimensional data set (Ringnér, 2008). Therefore, we performed PCA of the transcriptome data to illustrate the overall expression pattern of the six transgenic HR lines. The PCA score plot showed apparent clustering of the RNAi lines with the same silenced gene and separation of the RNAi lines with the different silenced genes across PC1. Although ERF1i-7 and ERF1i-19 were separated along PC2, they were in a different quartile from ERF2i and GUSi (Figure 4). This clustering and separation pattern suggested that, the transcriptome profile resulting from silencing of AP2/ERFs showed a specific perturbation response. To obtain more details of the transcripts in response to the silencing of *OpERF1* and *OpERF2*, differential expression analysis of the suppressed *OpERF* lines and GUSi lines was performed using DESeq2 (Love et al., 2014). Overall, 291 of 907 upregulated contigs and 112 of 450 downregulated contigs were differentially expressed in both ERF1i-7 and ERF1i-19. A higher number of the perturbed transcripts were found in ERF2i lines; 1180 of 1591 upregulated contigs and 352 of 691 downregulated contigs were differentially expressed in both ERF2i-2 and ERF2i-3 (Table 3). The higher number of affected transcripts in ERF2i than in ERF1i inferred that the expression of more genes was affected by the suppression of *OpERF2* and that the overlap of perturbed transcripts was higher between ERF2i-2 and ERF2i-3 than between ERF1i-7 and ERF1i-19. This observation was also demonstrated by the position of the samples in the PCA score plot, in which ERF2i-2 and ERF2i-3 were grouped together but ERF1i-7 and ERF1i-19 were separated (Figure 4).

**Table 3:** **Differentially expressed transcripts in ERF1i and ERF2i compared with average expression in GUSi**.

**Differentially expressed transcripts**	**Upregulated**	**Downregulated**
**All differentially expressed transcripts in ERF1i**	**907**	**450**
Both ERF1i-7 and ERF1i-19	291	112
Only in ERF1i-7	213	70
Only in ERF1i-19	403	268
**All differentially expressed transcripts in ERF2i**	**1591**	**691**
Both ERF2i-2 and ERF2i-3	1180	352
Only in ERF2i-2	118	68
Only in ERF2i-3	293	271

### Effects of *OpERF* suppression on the MEP and secologanin-strictosidine pathways

**Figure 5:**
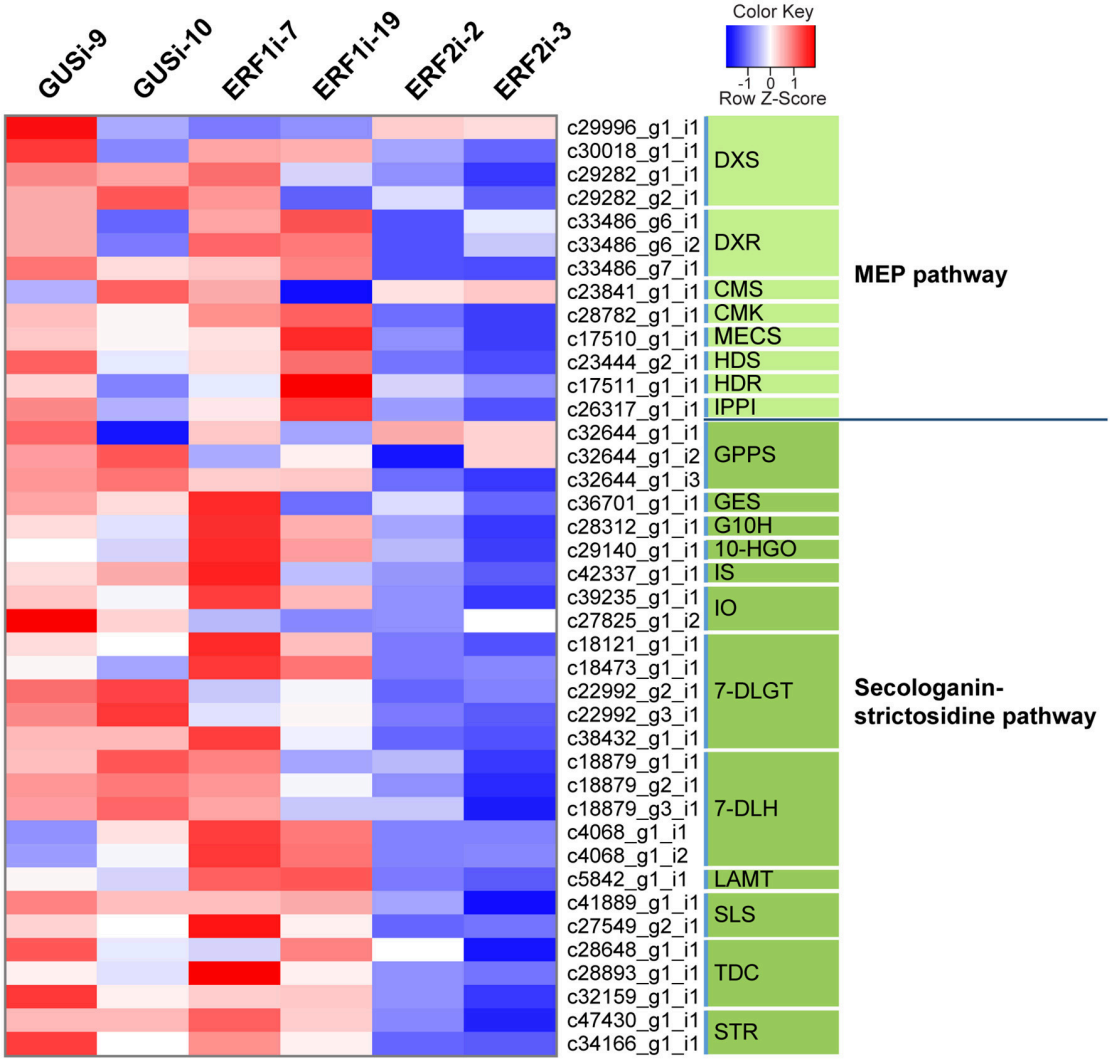
**Heatmap diagram of expression levels of genes in MEP and secologanin-strictosidine pathways**. The heatmap was constructed using FPKM of the putative MEP and secologanin-strictosidine pathways genes. The expression levels are illustrated in blue-red scale. Blue indicates lower expression and red indicates higher expression. Enzyme genes and their corresponding contigs are listed on the right side. DXS, 1-deoxy-d-xylulose-5-phosphate synthase; DXR, 1-deoxy-d-xylulose-5-phosphate reductoisomerase; CMS, 4-diphosphocytidyl-methylerythritol 2-phosphate synthase; CMK, 4-diphosphocytidyl-2-*C*-methyl-d-erythritol kinase; MECS, 2*C*-methyl-d-erythritol 2,4-cyclodiphosphate synthase; HDS, 4-hydroxy-3-methylbut-2-en-1-yl diphosphate synthase; HDR, 1-hydroxy-2-methyl-butenyl 4-diphosphate reductase, IPPI; isopentenyl diphosphate isomerase; GPPS, geranyl diphosphate synthase; GES, geraniol synthase; G10H, geraniol 10-hydroxylase; 10-HGO, 10-hydroxygeraniol oxidoreductase; IS, iridoid synthase; IO, iridoid oxidase; 7-DLGT, 7-deoxyloganetic acid glucosyl transferase; 7-DLH, 7-deoxyloganic acid hydroxylase; LAMT, loganic acid *O*-methyltransferase; SLS, secologanin synthase; TDC, tryptophan decarboxylase; STR, strictosidine synthase.

**Figure 6:**
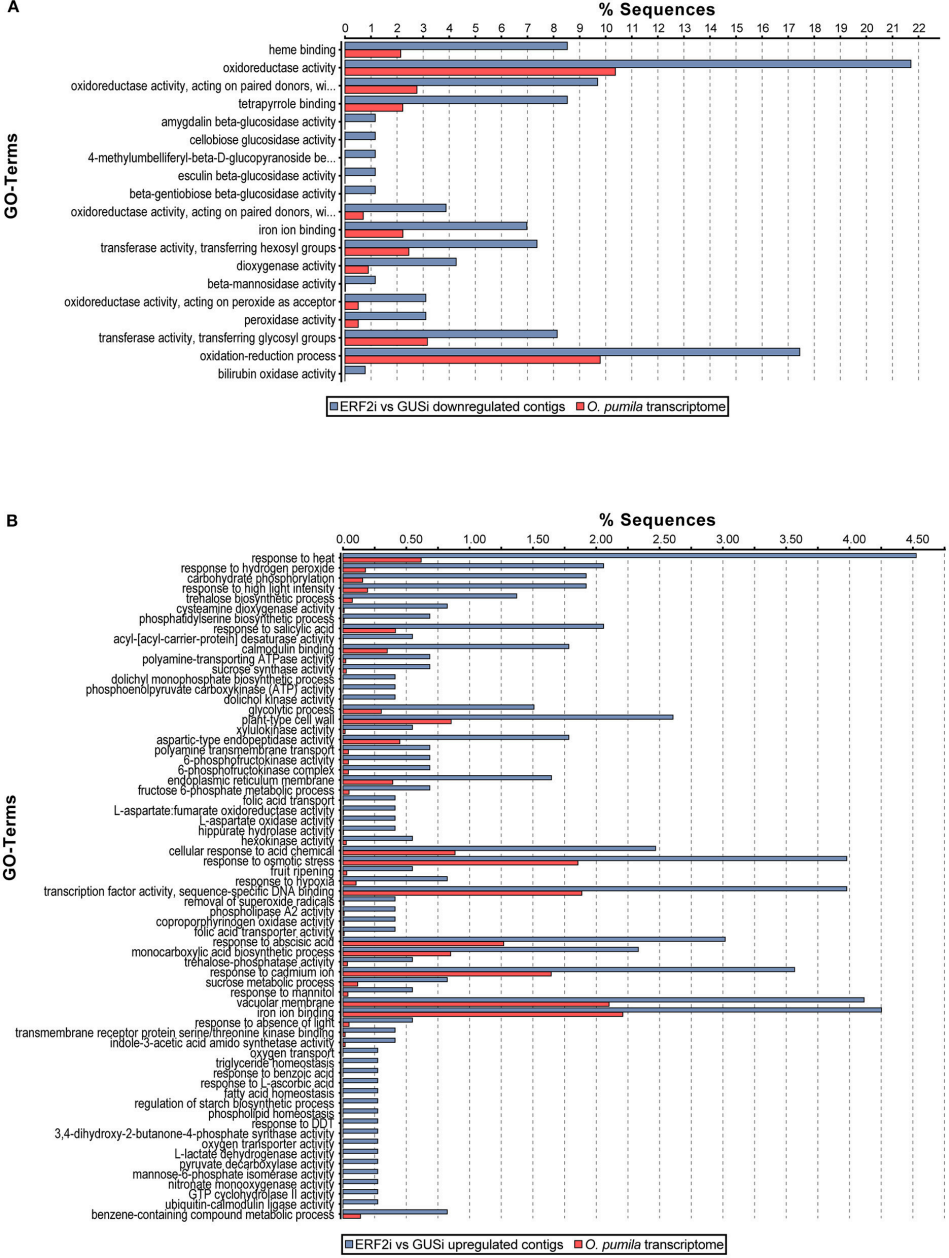
**Gene ontology enrichment of differentially expressed transcripts in ERF2i**. GO enrichment analysis of **(A)** downregulated and **(B)** upregulated transcripts in ERF2i compare to GUSi was performed using Fisher's exact test with a false discovery rate cut-off 0.05. Percentage of enriched GO terms statistically significant with respect to a reference set of *O. pumila de novo* transcriptome assembly are shown.

Previous studies on the involvement of the group IX ERF factors in alkaloid biosynthesis in several plants increased our interest to evaluate whether *Op*ERF2 has a similar function because it has a close phylogenetic relationship with secondary metabolite-regulating group IX ERFs. We investigated the expression levels of transcripts in the MEP pathway that synthesizes isopentenyl pyrophosphate and dimethylallyl pyrophosphate, which are primary metabolite substrates of TIA biosynthesis in plant, and secologanin-strictosidine pathway that produces strictosidine, a precursor of CPT (Yamazaki et al., 2004) and other TIAs in *O. pumila*. Putative MEP and secologanin-strictosidine pathways transcripts in *O. pumila* were identified on the basis of sequence similarity with the corresponding reference genes from *C. roseus* (Table S2). In a heatmap representing the comparative degree of expression between RNAi lines, a trend of downregulation of MEP and secologanin-strictosidine pathways genes was observed when *OpERF2* was suppressed; however this trend was not observed in ERF1i lines (Figure 5). In addition, expression levels of four characterized *O. pumila* genes (*OpTDC, OpG10H, OpSLS*, and *OpSTR*) in the transgenic HR lines determined using qRT-PCR were evaluated to affirm the presumption made through RNA-seq based transcriptome profiling. The qRT-PCR results showed a similar trend of gene expression with the transcriptome data, with a moderate degree of structural gene suppression in ERF2i (Figure S4). We further conducted GO enrichment analysis of the differentially expressed transcripts to illustrate the functional properties enriched in the transcript set of ERF2i lines by using Fisher's exact test against the annotated *de novo* transcriptome assembly of *O. pumila*. Consistent with the observation made using qRT-PCR and heatmap, GO terms associated with oxidation-reduction that are generally involved in secondary metabolite biosynthesis were enriched in the downregulated transcripts of the ERF2i lines. In the ERF2i upregulated transcript set, a broad range of enriched GO terms related to primary metabolism was enlisted (Figure 6).

To examine the effects of reduced *OpERF* expression on the metabolomic profile of transgenic HR lines, untargeted metabolome analysis of six RNAi lines (six biological replicates per line) was performed using LC-QTOF-MS. Chemical assignment to ion peaks was conducted based on reference *m/z* values as reported by Yamazaki et al. (2013), and metabolite levels were determined using peak intensity. Despite a trend of reduced expression of CPT precursor-producing enzyme genes in ERF2i, no drastic difference in the accumulation of CPT and its late-step intermediates was observed among the RNAi lines (Figure S5). To further understand the differences in the metabolic profiles of the RNAi lines, PCA was conducted using the normalized intensities of all 3735 detected peaks. The PCA score plot showed clustering of ERF2i, clustering GUSi, and dissociation of ERF1i-7 and ERF1i-19 at a short distance. Proximity of the samples in the PCA score plot as well indicated no drastic difference in the metabolomic profiles (Figure S6).

In this study, a trend of downregulation was observed throughout MIA precursor biosynthesis pathways, starting from the MEP pathway to the production of strictosidine in response to *OpERF2* suppression. In *C. roseus*, the regulation of early MIA biosynthesis is reportedly being separated between two families of transcription factors. ERF transcription factors, ORCA2 and ORCA3, only act on late secologanin-strictosidine pathway (*TDC, loganicacid-O-methyltransferase, SLS*, and *STR*) and *strictosidine-β-glucosidase* (Menke et al., 1999; van der Fits and Memelink, 2000, 2001; Miettinen et al., 2014). On the other hand, the basic helix-loop-helix (bHLH) transcription factors, BIS1 and BIS2, specifically regulate the genes in MEP and iridoid pathways (Van Moerkercke et al., 2015, 2016). This demonstrates a diversity in transactivation patterns between homologs from difference species.

Several studies have reported a basic helix-loop-helix transcription factor MYC2 acting upstream of group IX ERFs in response to the jasmonate signal to regulate synthesis of specialized metabolism. In *C. roseus*, MYC2 binds to the jasmonate-responsive element in the promoter region of *ORCA3* and can induce *ORCA3* expression, which then results in increased TIA levels (Zhang et al., 2011). In tobacco and tomato, MYC2 regulate nicotine alkaloids and cholesterol biosynthesis genes, respectively, by induction of group IX ERFs expression, acting synergistically with group IX ERFs, or binding directly to alkaloid biosynthesis genes (Shoji and Hashimoto, 2011; Cárdenas et al., 2016). We expected either no alteration of *MYC2* expression or upregulation of *MYC2* to compensate the function of *OpERF2* in *ERF2* suppressed lines. Surprisingly, the levels of putative *MYC2* transcripts were slightly downregulated in ERF2i (log_2_ FPKM fold change −0.4 to −0.6 in ERF2i when compared with GUSi). Since the available data are not substantial to make a conclusion, further study is required to confirm the relationship between ERF2 and MYC2 in *O. pumila*.

The inconsistency between enzyme expression and metabolite accumulation is not unexpected, as observed in the study conducted by Peebles et al. (2009). Transgenic HR of *C. roseus* that overexpressed *ORCA3* failed to increase the accumulation of TIAs, despite induced expression of key enzyme genes in TIA biosynthesis. Moreover, a complex biosynthesis network for secondary metabolites may also avert the effects of suppressed *OpERF*s on the alteration of CPT and intermediates in HR. Therefore, besides alteration of transcription factor expression, other measures such as inhibition of metabolic flux to branching pathways, escalation of enzyme activity in rate-limiting steps, and augmentation of metabolite transportation and metabolic sink should also be considered for the successful enhancement of CPT production.

### Jasmonic acid-related GO annotation in the ERF1i lines

As discussed in an earlier section that transcript levels of MEP and secologanin-strictosidine pathways were not affected in ERF1i, we examined enriched GO terms in ERF1i to understand the scope of categories affected by *OpERF1* suppression. In the ERF1i upregulated transcript set, several stress response-related GO terms, including jasmonic acid response, were enriched (Figure S7). Jasmonic acid is a phytohormone involved in various biological processes in plants, especially in wounding and defense responses. Therefore, we evaluated the expression profiles of enzyme genes in jasmonic acid biosynthesis by using Arabidopsis genes as a reference for the identification of putative genes in *O. pumila* (Table S2). A heatmap generated using FPKM of the corresponding contigs showed only moderately induced expression in ERF1i-19, suggesting no effect on jasmonic acid biosynthesis in response to the suppression of *OpERF1* (Figure S8). Arabidopsis group VII ERFs have been confirmed to respond to a low-oxygen environment and biotic stress; hence, the enrichment of several stress-associated GO terms in ERF1i upregulated genes suggested that *OpERF1* may be responsible for a similar function. Further studies on these stress responses are required to validate this hypothesis.

## Conclusion

To the best of our knowledge, this is the first report on the functional characterization of AP2/ERF transcription factors associated with an important specialized metabolism in *O. pumila*. We isolated *OpERF1* to *OpERF5*, five key AP2/ERF genes specially expressed in CPT-producing HR vs. non-CPT-producing CSC. On the basis of the transcriptome analysis and phylogenetic relationships with secondary metabolite-regulating AP2/ERFs, a dominantly expressed *OpERF1* and alkaloid-regulating AP2/ERF homolog *OpERF2* were selected for the functional characterization. We generated the transgenic HR lines with suppressed expression of *OpERF1* or *OpERF2* and used the transcriptome and metabolome to identify the specialized metabolic pathways affected by the altered *OpERF1* and *OpERF2* expressions. The phylogenetic relationship of *Op*ERF1 with Arabidopsis group VII ERFs and enrichment of gene ontologies related to stress response in *OpERF1*-suppressed lines suggested its function during stress conditions. The positive role of *Op*ERF2 in regulation of secondary metabolism was demonstrated in ERF2i lines. Suppression of *OpERF2* resulted in the downregulation of genes in the MEP and secologanin-strictosidine pathways, which are early steps that supply a precursor for CPT biosynthesis in *O. pumila*; however, effects on metabolite accumulation were not observed. The finding of this study serve as an accentuating reference for a future study on the regulators of valuable alkaloid production in other medicinal plants and a model for the sustainable production of commercially important crops and herbs.

## Author contributions

KS, MY: conceived and designed the experiments. NU, JO, and RI: performed the cloning of *OpERF*s. NU: conducted sequence analysis, RNAi, laboratory analysis, and data interpretation. HS: performed deep-transcriptome sequencing. AR: performed transcriptome analysis. TM, RN: performed untargeted metabolome analysis. NU, AR, MY, and KS: contributed to the manuscript preparation.

## Funding

This work is supported in part by Grants-in-Aid for Scientific Research (KAKENHI) from The Ministry of Education, Culture, Sports, Science, and Technology (MEXT), and by Strategic Priority Research Promotion Program, Chiba University.

### Conflict of interest statement

The authors declare that the research was conducted in the absence of any commercial or financial relationships that could be construed as a potential conflict of interest. The reviewer YY and handling Editor declared their shared affiliation, and the handling Editor states that the process nevertheless met the standards of a fair and objective review.

## Supplementary material

Figure S1 **Schematic diagram of (A)** T-DNA region of pGWB80 vector used in RNAi experiments (not drawn to scale), **(B)** *OpERF1* and *OpERF2* sequences. Open reading frame is represented by a white box and AP2/ERF domain is represented by a black box. Solid lines and dashed lines under *OpERF* sequences indicate the part of genes used as the target sequences in binary vector construction and the part of genes amplified in qRT-PCR, respectively. RB, right border; LB, left border; Km-r, kanamycin resistant gene; 35S-P, Cauliflower Mosaic Virus 35S promoter; NOS-T, nos terminator; Hyg-r, hygromycin resistant gene; bp, base pair.

Figure S2 **Amino acid sequence alignment of (A)** *Op*ERF1 to *Op*ERF5, **(B)** *Op*ERF1, and group VII Arabidopsis AP2/ERFs Op, *Ophiorrhiza pumila*; At, *Arabidopsis thaliana*. The sequences were aligned using ClustalW in Bioedit software version 7.2.5. AP2/ERF domain is enclosed in a black box. N-terminal MCGGAI(I/L) motif is enclosed in a red box. Asterisks indicate the reserved amino acids at position 14 and 19 of AP2/ERF domain. Solid bar indicates WLG motif.

Figure S3 **Expression levels of *OpERF1* and *OpERF2* in RNAi lines**. Data represents means of 3 repeated experiments (*n* = 1) ± standard deviation.

Figure S4 **Expression levels of MIA biosynthesis genes in RNAi lines**. Expression fold change relative to GUSi determined using qRT-PCR is presented by bar graph. FPKM of enzyme's corresponding contig obtained from RNA-seq is presented by line graph. qRT-PCR data represents means of 4 biological replicates ± standard deviation and experiments were repeated twice. Asterisks indicate significant differences compared to average of GUSi (*t*-test, ^*^*p* < 0.05, ^**^*p* < 0.01).

Figure S5 **Accumulation levels of CPT and intermediates in RNAi lines**. Data represents means of 6 biological replicates ± standard deviation. Asterisks indicate significant differences compared to average of GUSi (*t*-test, ^*^*p* < 0.05, ^**^*p* < 0.01).

Figure S6 **PCA score plot generated from metabolome data of RNAi lines**.

Figure S7 **Gene ontology enrichment of differentially expressed transcripts in ERF1i**. GO enrichment analysis of **(A)** upregulated and **(B)** downregulated transcripts in ERF1i compare to GUSi was performed using Fisher's exact test with a false discovery rate cut-off 0.05. Percentage of enriched GO terms statistically significant with respect to a reference set of *O. pumila de novo* transcriptome assembly are shown.

Figure S8 **Heatmap diagram of expression levels of genes in jasmonic acid biosynthesis pathway**. The heatmap was constructed using FPKM of the putative jasmonic acid biosynthetic genes. The expression levels are illustrated in blue-red scale. Blue indicates lower expression and red indicates higher expression. Enzyme genes and their corresponding contigs are listed on the right side. LOX, linoleate 13*S*-lipoxygenase; AOS, allene oxide synthase; AOC, allene oxide cyclase; OPR, 12-oxophytodienoate reductase; OPCL, OPC ligase; ACX, fatty acyl-CoA oxidase; AIM, enoyl-CoA hydratase/3-hydroxyacyl-CoA dehydrogenase; PKT, 3-keto-acyl-CoA-thiolase.

Table S1 **Primers used in qRT-PCR**.

Table S2 **FPKM of the putative enzyme contigs used in heatmaps**.

Table S3 **Annotation and expression level of unigenes with AP2/ERF domain highly expressed in *O. pumila* hairy roots**.

## References

[B1] Aimi N. Nishimura M. Miwa A. Hoshino H. Sakai S. Haginiwa J. (1989). Pumiloside and deoxypumiloside; plausible intermediates of camptothecin biosynthesis. Tetrahedron Lett. 30, 4991–4994. 10.1016/S0040-4039(01)80563-3

[B2] Amirkia V. Heinrich M. (2014). Alkaloids as drug leads – A predictive structural and biodiversity-based analysis. Phytochem. Lett. 10, xlviii–liii. 10.1016/j.phytol.2014.06.015

[B3] Asano T. Kobayashi K. Kashihara E. Sudo H. Sasaki R. Iijima Y.. (2013). Suppression of camptothecin biosynthetic genes results in metabolic modification of secondary products in hairy roots of *Ophiorrhiza pumila*. Phytochemistry 91, 128–139. 10.1016/j.phytochem.2012.04.019 22652243

[B4] Bakkali F. Averbeck S. Averbeck D. Idaomar M. (2008). Biological effects of essential oils – A review. Food Chem. Toxicol. 46, 446–475. 10.1016/j.fct.2007.09.106 17996351

[B5] Bunsupa S. Okada T. Saito K. Yamazaki M. (2011). An acyltransferase-like gene obtained by differential gene expression profiles of quinolizidine alkaloid-producing and nonproducing cultivars of *Lupinus angustifolius*. Plant Biotechnol. 28, 89–94. 10.5511/plantbiotechnology.10.1109b

[B6] Cárdenas P. D. Sonawane P. D. Pollier J. Vanden Bossche R. Dewangan V. Weithorn E.. (2016). GAME9 regulates the biosynthesis of steroidal alkaloids and upstream isoprenoids in the plant mevalonate pathway. Nat. Commun. 7:10654. 10.1038/ncomms10654 26876023 PMC4756317

[B7] Chuck G. Meeley R. B. Hake S. (1998). The control of maize spikelet meristem fate by the *APETALA2*-like gene indeterminate spikelet1. Genes Dev. 12, 1145–1154. 10.1101/gad.12.8.1145 9553044 PMC316712

[B8] Coleman R. L. (2002). Emerging role of topotecan in front-line treatment of carcinoma of the ovary. Oncologist 7, 46–55. 10.1634/theoncologist.7-suppl_5-46 12324633

[B9] Croteau R. Kutchan T. M. Lewis N. G. (2000). Natural products (secondary metabolites), in Biochemistry & *Molecular Biology of Plants*, eds Buchanan B. Gruissem W. Jones R.(Rockville, MD: American Society of Plant Physiologists), 1250–1318.

[B10] Cunningham D. Maroun J. Vanhoefer U. Cutsem E. V. (2001). Optimizing the use of irinotecan in colorectal cancer. Oncologist 6, 17–23. 10.1038/nrclinonc.2009.140 11585970

[B11] Edgar R. Domrachev M. Lash A. E. (2002). Gene expression omnibus: NCBI gene expression and hybridization array data repository. Nucleic Acids Res. 30, 207–210. 10.1093/nar/30.1.207 11752295 PMC99122

[B12] Fischer U. Dröge-Laser W. (2004). Overexpression of *NtERF5*, a new member of the tobacco ethylene response transcription factor family enhances resistance to tobacco mosaic virus. Mol. Plant. Microbe Interact. 17, 1162–1171. 10.1094/MPMI.2004.17.10.1162 15497409

[B13] Garst J. (2007). Topotecan: an evolving option in the treatment of relapsed small cell lung cancer. Ther. Clin. Risk Manag. 3, 1087–1095. 18516270 PMC2387299

[B14] Govindachari T. R. Viswanathan N. (1972). Alkaloids of *Mappia foetida*. Phytochemistry 11, 3529–3531. 10.1016/S0031-9422(00)89852-0

[B15] Hall T. (1999). BioEdit: a user-friendly biological sequence alignment editor and analysis program for Windows 95/98/NT. Nucleic Acids Symp. Ser. 41, 95–98.

[B16] Hsiang Y. H. Hertzberg R. Hecht S. Liu L. F. (1985). Camptothecin induces protein-linked DNA breaks via mammalian DNA topoisomerase I. J. Biol. Chem. 260, 14873–14878. 2997227

[B17] Hsieh E. J. Cheng M. C. Lin T. P. (2013). Functional characterization of an abiotic stress-inducible transcription factor AtERF53 in *Arabidopsis thaliana*. Plant Mol. Biol. 82, 223–237. 10.1007/s11103-013-0054-z 23625358

[B18] Jacobo-Velázquez D. A. González-Agüero M. Cisneros-Zevallos L. (2015). Cross-talk between signaling pathways: the link between plant secondary metabolite production and wounding stress response. Sci. Rep. 5:8608. 10.1038/srep08608 25712739 PMC5390084

[B19] Jin J. Zhang H. Kong L. Gao G. Luo J. (2014). PlantTFDB 3.0: a portal for the functional and evolutionary study of plant transcription factors. Nucleic Acids Res. 42, D1182–D1187. 10.1093/nar/gkt1016 24174544 PMC3965000

[B20] Kim Y. M. Lee S. W. Kim D. Y. Kim J. H. Nam J. H. Kim Y. T. (2010). The efficacy and toxicity of belotecan (CKD-602), a camptothecin analogue topoisomerase I inhibitor, in patients with recurrent or refractory epithelial ovarian cancer. J. Chemother. 22, 197–200. 10.1179/joc.2010.22.3.197 20566426

[B21] Krishna P. (2003). Brassinosteroid-mediated stress responses. J. Plant Growth Regul. 22, 289–297. 10.1007/s00344-003-0058-z 14676968

[B22] Langmead B. Trapnell C. Pop M. Salzberg S. L. (2009). Ultrafast and memory-efficient alignment of short DNA sequences to the human genome. Genome Biol. 10:R25. 10.1186/gb-2009-10-3-r25 19261174 PMC2690996

[B23] Larkin M. A. Blackshields G. Brown N. P. Chenna R. McGettigan P. A. McWilliam H.. (2007). Clustal W and Clustal X version 2.0. Bioinformatics 23, 2947–2948. 10.1093/bioinformatics/btm404 17846036

[B24] Li B. Dewey C. N. (2011). RSEM: accurate transcript quantification from RNA-Seq data with or without a reference genome. BMC Bioinformatics 12:323. 10.1186/1471-2105-12-323 21816040 PMC3163565

[B25] Licausi F. Van Dongen J. T. Giuntoli B. Novi G. Santaniello A. Geigenberger P.. (2010). HRE1 and HRE2, two hypoxia-inducible ethylene response factors, affect anaerobic responses in *Arabidopsis thaliana*. Plant J. 62, 302–315. 10.1111/j.1365-313X.2010.04149.x 20113439

[B26] Lin R. C. Park H. J. Wang H. Y. (2008). Role of Arabidopsis RAP2.4 in regulating light- and ethylene-mediated developmental processes and drought stress tolerance. Mol. Plant 1, 42–57. 10.1093/mp/ssm004 20031913

[B27] Liu Q. Kasuga M. Sakuma Y. Abe H. Miura S. Yamaguchi-Shinozaki K.. (1998). Two transcription factors, DREB1 and DREB2, with an EREBP/AP2 DNA binding domain separate two cellular signal transduction pathways in drought- and low-temperature-responsive gene expression, respectively, in Arabidopsis. Plant Cell 10, 1391–1406. 10.1105/tpc.10.8.1391 9707537 PMC144379

[B28] Lorence A. Medina-Bolivar F. Nessler C. L. (2004). Camptothecin and 10-hydroxycamptothecin from *Camptotheca acuminata*. Plant Cell Rep. 22, 437–441. 10.1007/s00299-003-0708-4 13680137

[B29] Lorence A. Nessler C. L. (2004). Camptothecin, over four decades of surprising findings. Phytochemistry 65, 2735–2749. 10.1016/j.phytochem.2004.09.001 15474560

[B30] Love M. I. Huber W. Anders S. (2014). Moderated estimation of fold change and dispersion for RNA-seq data with DESeq2. Genome Biol. 15, 550. 10.1186/s13059-014-0550-8 25516281 PMC4302049

[B31] Lu X. Zhang L. Zhang F. Jiang W. Shen Q. Zhang L.. (2013). AaORA, a trichome-specific AP2/ERF transcription factor of *Artemisia annua*, is a positive regulator in the artemisinin biosynthetic pathway and in disease resistance to *Botrytis cinerea*. New Phytol. 198, 1191–1202. 10.1111/nph.12207 23448426

[B32] Menke F. L. Champion A. Kijne J. W. Memelink J. (1999). A novel jasmonate- and elicitor-responsive element in the periwinkle secondary metabolite biosynthetic gene *Str* interacts with a jasmonate- and elicitor-inducible AP2-domain transcription factor, ORCA2. EMBO J. 18, 4455–4463. 10.1093/emboj/18.16.4455 10449411 PMC1171520

[B33] Miettinen K. Dong L. Navrot N. Schneider T. Burlat V. Pollier J.. (2014). The seco-iridoid pathway from *Catharanthus roseus*. Nat. Commun. 5, 3606. 10.1038/ncomms4606 24710322 PMC3992524

[B34] Mithöfer A. Boland W. (2012). Plant defense against herbivores: chemical aspects. Annu. Rev. Plant Biol. 63, 431–450. 10.1146/annurev-arplant-042110-103854 22404468

[B35] Mizoi J. Shinozaki K. Yamaguchi-Shinozaki K. (2012). AP2/ERF family transcription factors in plant abiotic stress responses. Biochim. Biophys. Acta 1819, 86–96. 10.1016/j.bbagrm.2011.08.004 21867785

[B36] Munster P. N. Daud A. I. (2011). Preclinical and clinical activity of the topoisomerase I inhibitor, karenitecin, in melanoma. Expert Opin. Investig. Drugs 20, 1565–1574. 10.1517/13543784.2011.617740 21985236

[B37] Nakabayashi R. Yonekura-Sakakibara K. Urano K. Suzuki M. Yamada Y. Nishizawa T.. (2014). Enhancement of oxidative and drought tolerance in Arabidopsis by overaccumulation of antioxidant flavonoids. Plant J. 77, 367–379. 10.1111/tpj.12388 24274116 PMC4282528

[B38] Nakano T. Suzuki K. Fujimura T. Shinshi H. (2006). Genome-wide analysis of the ERF gene family in Arabidopsis and rice. Plant Physiol. 140, 411–432. 10.1104/pp.105.073783 16407444 PMC1361313

[B39] Ohto M. Floyd S. K. Fischer R. L. Goldberg R. B. Harada J. J. (2009). Effects of APETALA2 on embryo, endosperm, and seed coat development determine seed size in Arabidopsis. Sex. Plant Reprod. 22, 277–289. 10.1007/s00497-009-0116-1 20033449 PMC2796121

[B40] Oñate-Sánchez L. Singh K. B. (2002). Identification of Arabidopsis ethylene-responsive element binding factors with distinct induction kinetics after pathogen infection. Plant Physiol. 128, 1313–1322. 10.1104/pp.010862 11950980 PMC154259

[B41] Peebles C. A. Hughes E. H. Shanks J. V. San K.-Y. (2009). Transcriptional response of the terpenoid indole alkaloid pathway to the overexpression of ORCA3 along with jasmonic acid elicitation of *Catharanthus roseus* hairy roots over time. Metab. Eng. 11, 76–86. 10.1016/j.ymben.2008.09.002 18955153

[B42] Rai A. Nakamura M. Takahashi H. Suzuki H. Saito K. Yamazaki M. (2016b). High-throughput sequencing and *de novo* transcriptome assembly of *Swertia japonica* to identify genes involved in the biosynthesis of therapeutic metabolites. Plant Cell Rep. 35, 2091–2111. 10.1007/s00299-016-2021-z 27378356

[B43] Rai A. Yamazaki M. Takahashi H. Nakamura M. Kojoma M. Suzuki H.. (2016a). RNA-seq transcriptome analysis of *Panax japonicus*, and its comparison with other *Panax* species to identify potential genes involved in the saponins biosynthesis. Front. Plant Sci. 7:481. 10.3389/fpls.2016.00481 27148308 PMC4828455

[B44] Reuben S. Rai A. Pillai B. V. Rodrigues A. Swarup S. (2013). A bacterial quercetin oxidoreductase QuoA-mediated perturbation in the phenylpropanoid metabolic network increases lignification with a concomitant decrease in phenolamides in Arabidopsis. J. Exp. Bot. 64, 5183–5194. 10.1093/jxb/ert310 24085580 PMC3830493

[B45] Riechmann J. L. Meyerowitz E. M. (1998). The AP2/EREBP family of plant transcription factors. Biol. Chem. 379, 633–646. 9687012 10.1515/bchm.1998.379.6.633

[B46] Ringnér M. (2008). What is principal component analysis? Nat. Biotechnol. 26, 303–304. 10.1038/nbt0308-303 18327243

[B47] Rohani E. R. Chiba M. Kawaharada M. Asano T. Oshima Y. Mitsuda N. (2016). An MYB transcription factor regulating specialized metabolisms in *Ophiorrhiza pumila*. Plant Biotechnol. 33, 1–9. 10.5511/plantbiotechnology.15.1117a

[B48] Sadre R. Magallanes-Lundback M. Pradhan S. Salim V. Mesberg A. Jones A. D. (2016). Metabolite diversity in alkaloid biosynthesis: a multi-lane (diastereomer) highway for camptothecin synthesis in *Camptotheca acuminata*. Plant Cell 28, 1926–1944. 10.1105/tpc.16.00193 27432874 PMC5006703

[B49] Saito K. Sudo H. Yamazaki M. Koseki-Nakamura M. Kitajima M. Takayama H. (2001). Feasible production of camptothecin by hairy root culture of *Ophiorrhiza pumila*. Plant Cell Rep. 20, 267–271. 10.1007/s002990100320

[B50] Sakuma Y. Liu Q. Dubouzet J. G. Abe H. Shinozaki K. Yamaguchi-Shinozaki K. (2002). DNA-binding specificity of the ERF/AP2 domain of Arabidopsis DREBs, transcription factors involved in dehydration- and cold-Inducible gene expression. Biochem. Biophys. Res. Commun. 290, 998–1009. 10.1006/bbrc.2001.6299 11798174

[B51] Schluttenhofer C. Pattanaik S. Patra B. Yuan L. (2014). Analyses of *Catharanthus roseus* and *Arabidopsis thaliana* WRKY transcription factors reveal involvement in jasmonate signaling. BMC Genomics 15:502. 10.1186/1471-2164-15-502 24950738 PMC4099484

[B52] Sears M. T. Zhang H. Rushton P. J. Wu M. Han S. Spano A. J.. (2013). NtERF32: a non-*NIC2* locus AP2/ERF transcription factor required in jasmonate-inducible nicotine biosynthesis in tobacco. Plant Mol. Biol. 84, 49–66. 10.1007/s11103-013-0116-2 23934400

[B53] Shoji T. Hashimoto T. (2011). Tobacco MYC2 regulates jasmonate-inducible nicotine biosynthesis genes directly and by way of the *NIC2*-locus ERF genes. Plant Cell Physiol. 52, 1117–1130. 10.1093/pcp/pcr063 21576194

[B54] Shoji T. Kajikawa M. Hashimoto T. (2010). Clustered transcription factor genes regulate nicotine biosynthesis in tobacco. Plant Cell 22, 3390–3409. 10.1105/tpc.110.078543 20959558 PMC2990138

[B55] Sudo H. Yamakawa T. Yamazaki M. Aimi N. Saito K. (2004). Bioreactor production of camptothecin by hairy root cultures of *Ophiorrhiza pumila*. Biotechnol. Lett. 24, 359–363. 10.1023/A:1014568904957

[B56] Suttipanta N. Pattanaik S. Kulshrestha M. Patra B. Singh S. K. Yuan L. (2011). The transcription factor CrWRKY1 positively regulates the terpenoid indole alkaloid biosynthesis in *Catharanthus roseus*. Plant Physiol. 157, 2081–2093. 10.1104/pp.111.181834 21988879 PMC3327198

[B57] Tamura K. Stecher G. Peterson D. Filipski A. Kumar S. (2013). MEGA6: molecular evolutionary genetics analysis version 6.0. Mol. Biol. Evol. 30, 2725–2729. 10.1093/molbev/mst197 24132122 PMC3840312

[B58] Thagun C. Imanishi S. Kudo T. Nakabayashi R. Ohyama K. Mori T.. (2016). Jasmonate-responsive ERF transcription factors regulate steroidal glycoalkaloid biosynthesis in tomato. Plant Cell Physiol. 57, 961–975. 10.1093/pcp/pcw067 27084593

[B59] Todd A. T. Liu E. Polvi S. L. Pammett R. T. Page J. E. (2010). A functional genomics screen identifies diverse transcription factors that regulate alkaloid biosynthesis in *Nicotiana benthamiana*. Plant J. 62, 589–600. 10.1111/j.1365-313X.2010.04186.x 20202168

[B60] Tournier B. Sanchez-Ballesta M. T. Jones B. Pesquet E. Regad F. Latché A.. (2003). New members of the tomato ERF family show specific expression pattern and diverse DNA-binding capacity to the GCC box element. FEBS Lett. 550, 149–154. 10.1016/S0014-5793(03)00757-9 12935902

[B61] van der Fits L. Memelink J. (2000). ORCA3, a jasmonate-responsive transcriptional regulator of plant primary and secondary metabolism. Science 289, 295–297. 10.1126/science.289.5477.295 10894776

[B62] van der Fits L. Memelink J. (2001). The jasmonate-inducible AP2/ERF-domain transcription factor ORCA3 activates gene expression via interaction with a jasmonate-responsive promoter element. Plant J. 25, 43–53. 10.1046/j.1365-313x.2001.00932.x 11169181

[B63] Van Moerkercke A. Fabris M. Pollier J. Baart G. J. Rombauts S. Hasnain G.. (2013). CathaCyc, a metabolic pathway database built from *Catharanthus roseus* RNA-seq data. Plant Cell Physiol. 54, 673–685. 10.1093/pcp/pct039 23493402

[B64] Van Moerkercke A. Steensma P. Gariboldi I. Espoz J. Purnama P. C. Schweizer F.. (2016). The basic helix-loop-helix transcription factor BIS2 is essential for monoterpenoid indole alkaloid production in the medicinal plant *Catharanthus roseus*. Plant J. 88, 3–12. 10.1111/tpj.13230 27342401

[B65] Van Moerkercke A. Steensma P. Schweizer F. Pollier J. Gariboldi I. Payne R.. (2015). The bHLH transcription factor BIS1 controls the iridoid branch of the monoterpenoid indole alkaloid pathway in *Catharanthus roseus*. Proc. Natl. Acad. Sci. U.S.A. 112, 8130–8135. 10.1073/pnas.1504951112 26080427 PMC4491741

[B66] Verma P. Mathur A. K. Khan S. A. Verma N. Sharma A. (2015). Transgenic studies for modulating terpenoid indole alkaloids pathway in *Catharanthus roseus*: present status and future options. Phytochem. Rev. 10.1007/s11101-015-9447-8. [Epub ahead of print].

[B67] Wall M. E. Wani M. C. Cook C. E. Palmer K. H. McPhail A. T. Sim G. A. (1966). Plant antitumor agents. I. The isolation and structure of camptothecin, a novel alkaloidal leukemia and tumor inhibitor from *Camptotheca acuminata*. J. Am. Chem. Soc. 88, 3888–3890. 10.1021/ja00968a057

[B68] Wiedenfeld H. Furmanowa M. Roeder E. Guzewska J. Gustowski W. (1997). Camptothecin and 10-hydroxycamptothecin in callus and plantlets of *Camptotheca acuminata*. Plant Cell Tissue Organ Cult. 49, 213–218. 10.1023/A:1005704429339

[B69] Yamazaki M. Mochida K. Asano T. Nakabayashi R. Chiba M. Udomsom N.. (2013). Coupling deep transcriptome analysis with untargeted metabolic profiling in *Ophiorrhiza pumila* to further the understanding of the biosynthesis of the anti-cancer alkaloid camptothecin and anthraquinones. Plant Cell Physiol. 54, 686–696. 10.1093/pcp/pct040 23503598 PMC3653139

[B70] Yamazaki Y. Kitajima M. Arita M. Takayama H. Sudo H. Yamazaki M.. (2004). Biosynthesis of camptothecin. *In silico* and *in vivo* tracer study from [1-13C]glucose. Plant Physiol. 134, 161–170. 10.1104/pp.103.029389 14657405 PMC316296

[B71] Yamazaki Y. Sudo H. Yamazaki M. Aimi N. Saito K. (2003b). Camptothecin biosynthetic genes in hairy roots of *Ophiorrhiza pumila*: cloning, characterization and differential expression in tissues and by stress compounds. Plant Cell Physiol. 44, 395–403. 10.1093/pcp/pcg051 12721380

[B72] Yamazaki Y. Urano A. Sudo H. Kitajima M. Takayama H. Yamazaki M.. (2003a). Metabolite profiling of alkaloids and strictosidine synthase activity in camptothecin producing plants. Phytochemistry 62, 461–470. 10.1016/S0031-9422(02)00543-5 12620359

[B73] Yu Z. X. Li J. X. Yang C. Q. Hu W. L. Wang L. J. Chen X. Y. (2012). The jasmonate-responsive AP2/ERF transcription factors AaERF1 and AaERF2 positively regulate artemisinin biosynthesis in *Artemisia annua* L. Mol. Plant 5, 353–365. 10.1093/mp/ssr087 22104293

[B74] Zhang H. Hedhili S. Montiel G. Zhang Y. Chatel G. Pré M.. (2011). The basic helix-loop-helix transcription factor CrMYC2 controls the jasmonate-responsive expression of the *ORCA* genes that regulate alkaloid biosynthesis in *Catharanthus roseus*. Plant J. 67, 61–71. 10.1111/j.1365-313X.2011.04575.x 21401746

[B75] Zhang H. Huang L. Dai Y. Liu S. Hong Y. Tian L.. (2015). Arabidopsis AtERF15 positively regulates immunity against *Pseudomonas syringae* pv. tomato DC3000 and *Botrytis cinerea*. Front. Plant Sci. 6:686. 10.3389/fpls.2015.00686 26388886 PMC4559647

[B76] Zhang J. Y. Broeckling C. D. Blancaflor E. B. Sledge M. K. Sumner L. W. Wang Z. Y. (2005). Overexpression of *WXP1*, a putative *Medicago truncatula* AP2 domain-containing transcription factor gene, increases cuticular wax accumulation and enhances drought tolerance in transgenic alfalfa (*Medicago sativa*). Plant J. 42, 689–707. 10.1111/j.1365-313X.2005.02405.x 15918883

[B77] Zhao Y. Wei T. Yin K. Q. Chen Z. Gu H. Qu L. J.. (2012). Arabidopsis RAP2.2 plays an important role in plant resistance to *Botrytis cinerea* and ethylene responses. New Phytol. 195, 450–460. 10.1111/j.1469-8137.2012.04160.x 22530619

