# Supplementary figures and images for: Function of AP2/ERF Transcription Factors Involved in the Regulation of Specialized Metabolism in Ophiorrhiza pumila Revealed by Transcriptomics and Metabolomics
Source: Front Plant Sci. 2016 Dec 9;7:1861. doi: 10.3389/fpls.2016.01861 (PMC5145908; doi:10.3389/fpls.2016.01861)

A

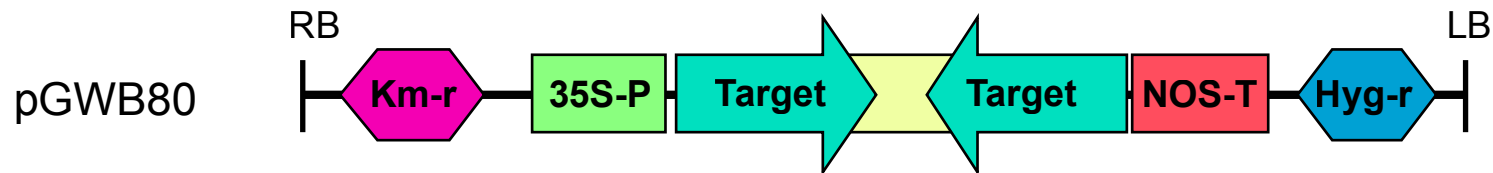

B

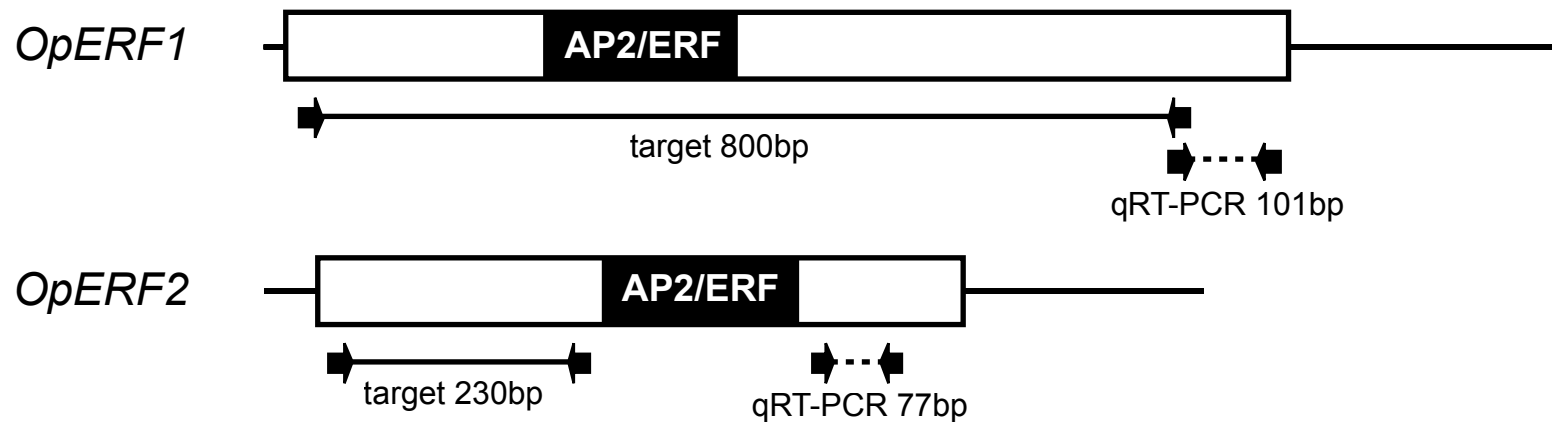

Supplement: Figure S1 — Schematic diagram of (A) T-DNA region of pGWB80 vector used in RNAi experiments (not drawn to scale), (B) OpERF1 and OpERF2 sequences. Open reading frame is represented by a white box and AP2/ERF domain is represented by a black box. Solid lines and dashed lines under OpERF sequences indicate the part of genes used as the target sequences in binary vector construction and the part of genes amplified in qRT-PCR, respectively. RB, right border; LB, left border; Km-r, kanamycin resistant gene; 35S-P, Cauliflower Mosaic Virus 35S promoter; NOS-T, nos terminator; Hyg-r, hygromycin resistant gene; bp, base pair. [file Image1.PDF]

*OpERF1*

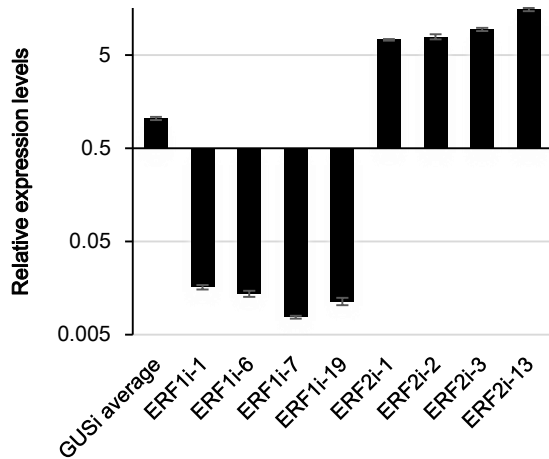

*OpERF2*

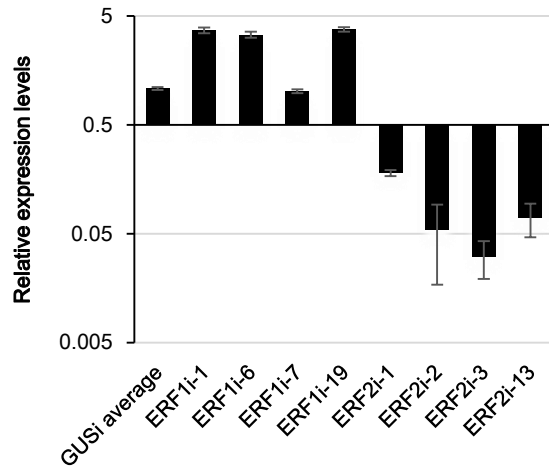

Supplement: Figure S3 — Expression levels of OpERF1 and OpERF2 in RNAi lines. Data represents means of 3 repeated experiments (n = 1) ± standard deviation. [file Image3.PDF]

*OpTDC*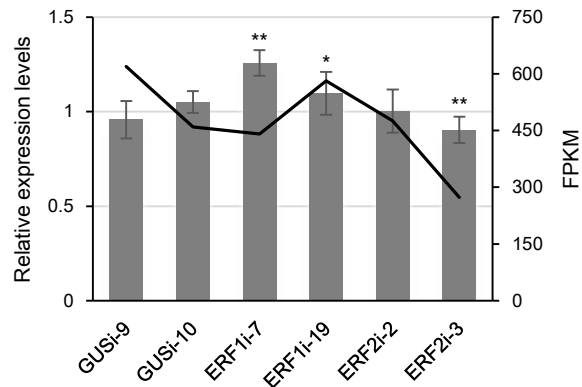*OpG10H*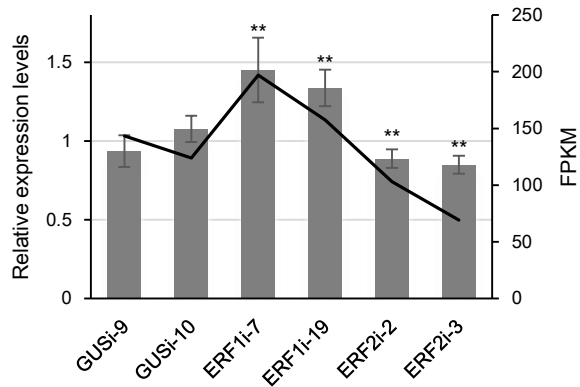*OpSLS*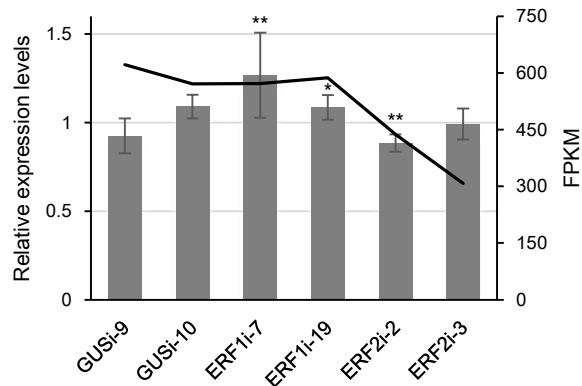*OpSTR*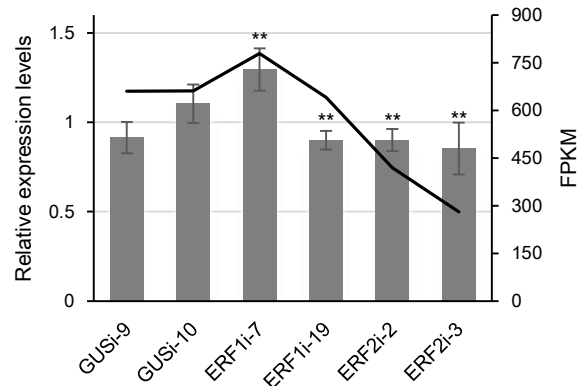

Supplement: Figure S4 — Expression levels of MIA biosynthesis genes in RNAi lines. Expression fold change relative to GUSi determined using qRT-PCR is presented by bar graph. FPKM of enzyme's corresponding contig obtained from RNA-seq is presented by line graph. qRT-PCR data represents means of 4 biological replicates ± standard deviation and experiments were repeated twice. Asterisks indicate significant differences compared to average of GUSi (t-test, *p < 0.05, **p < 0.01). [file Image4.PDF]

**Strictosidine**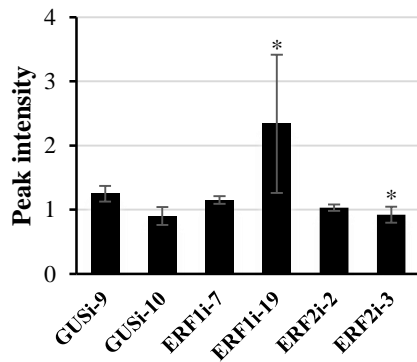**Strictosamide**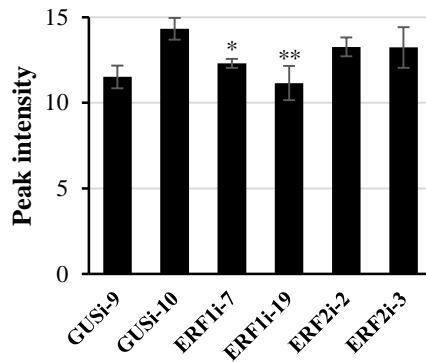**Pumiloside**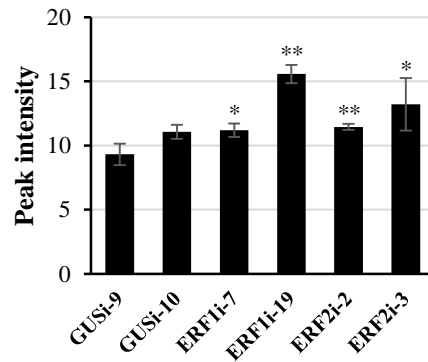**Deoxypumiloside1**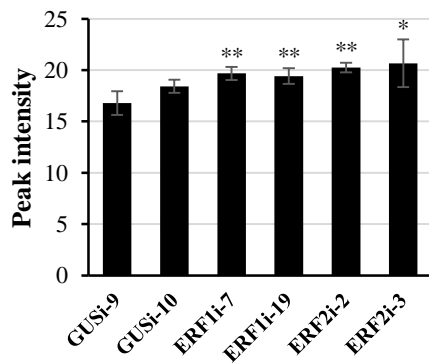**Deoxypumiloside2**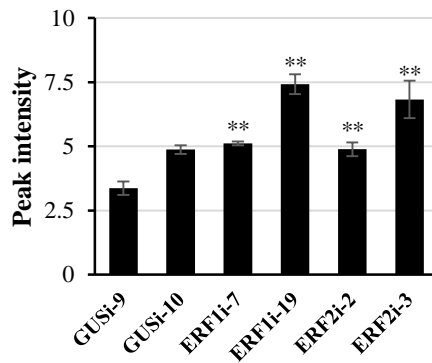**CPT**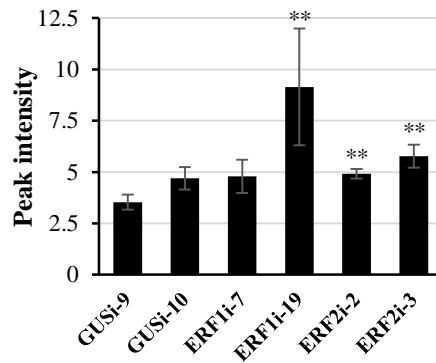

Supplement: Figure S5 — Accumulation levels of CPT and intermediates in RNAi lines. Data represents means of 6 biological replicates ± standard deviation. Asterisks indicate significant differences compared to average of GUSi (t-test, *p < 0.05, **p < 0.01). [file Image5.PDF]

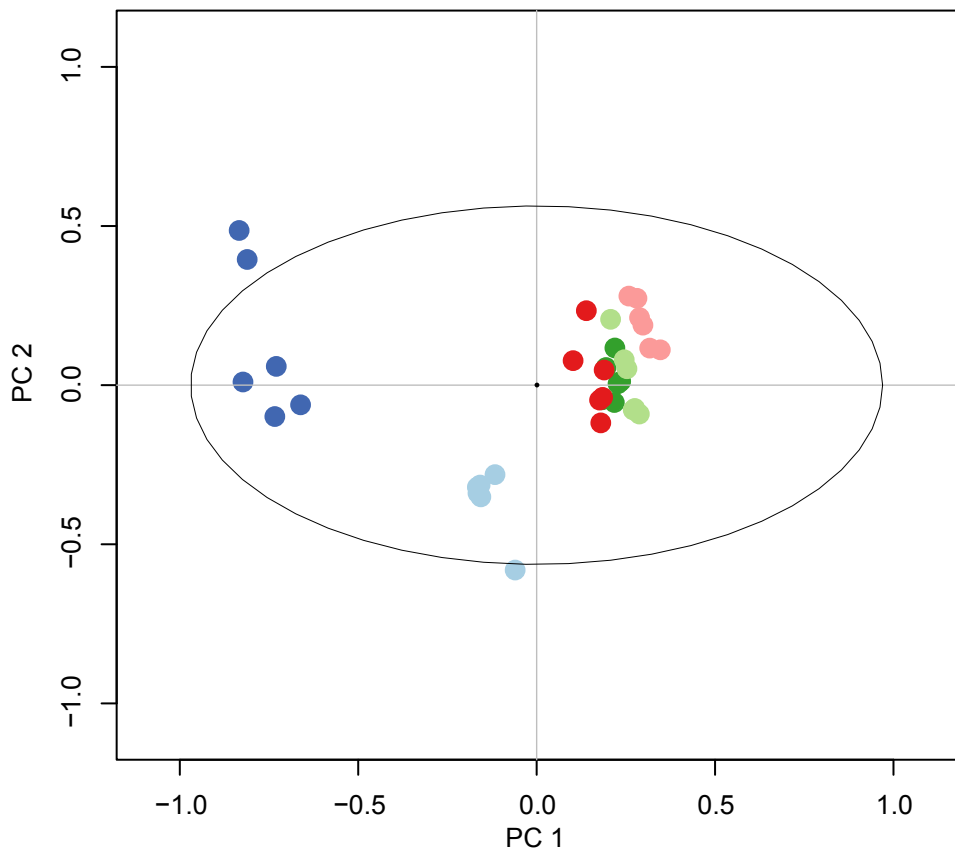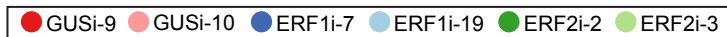

Supplement: Figure S6 — PCA score plot generated from metabolome data of RNAi lines. [file Image6.PDF]

**A**

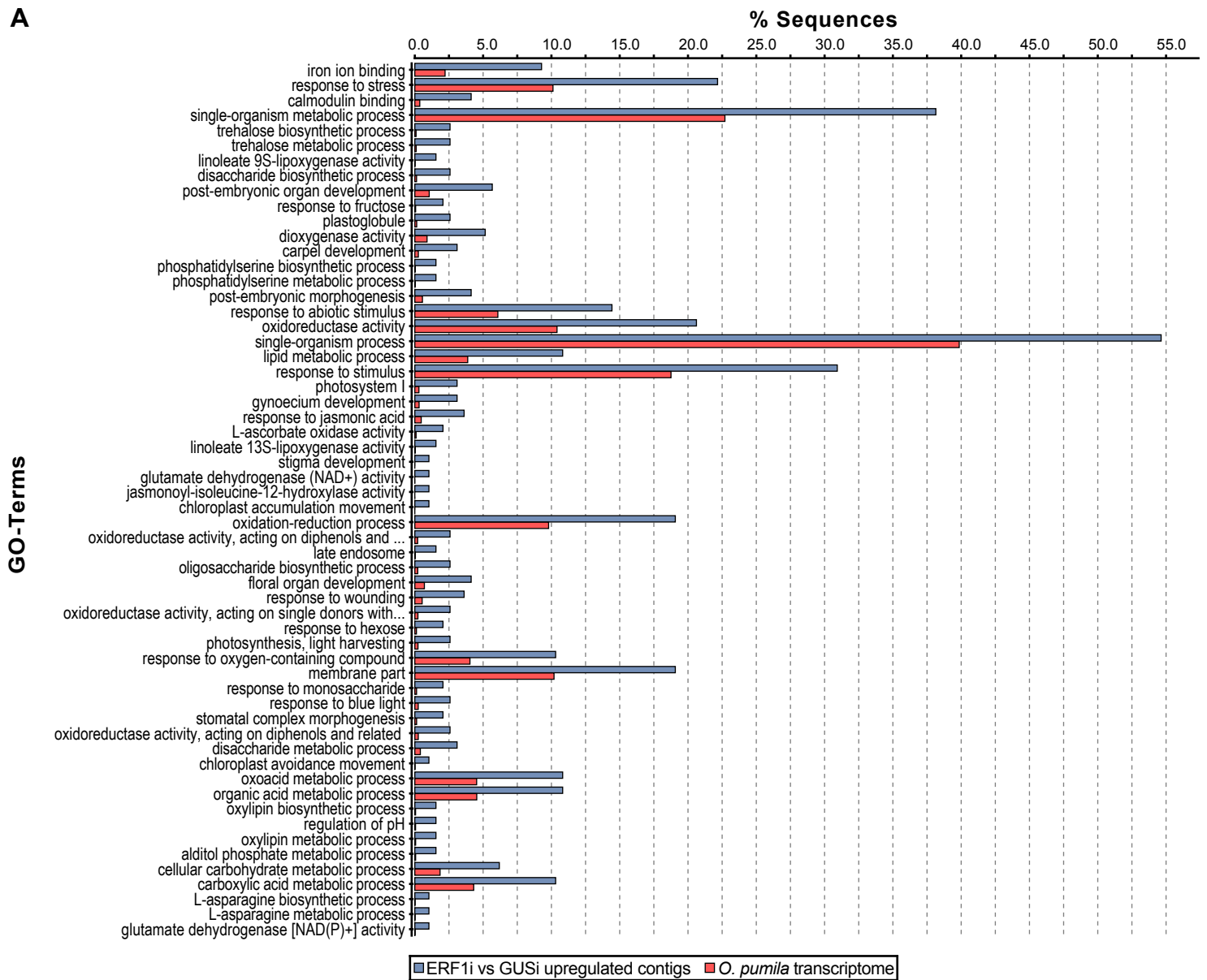

**B**

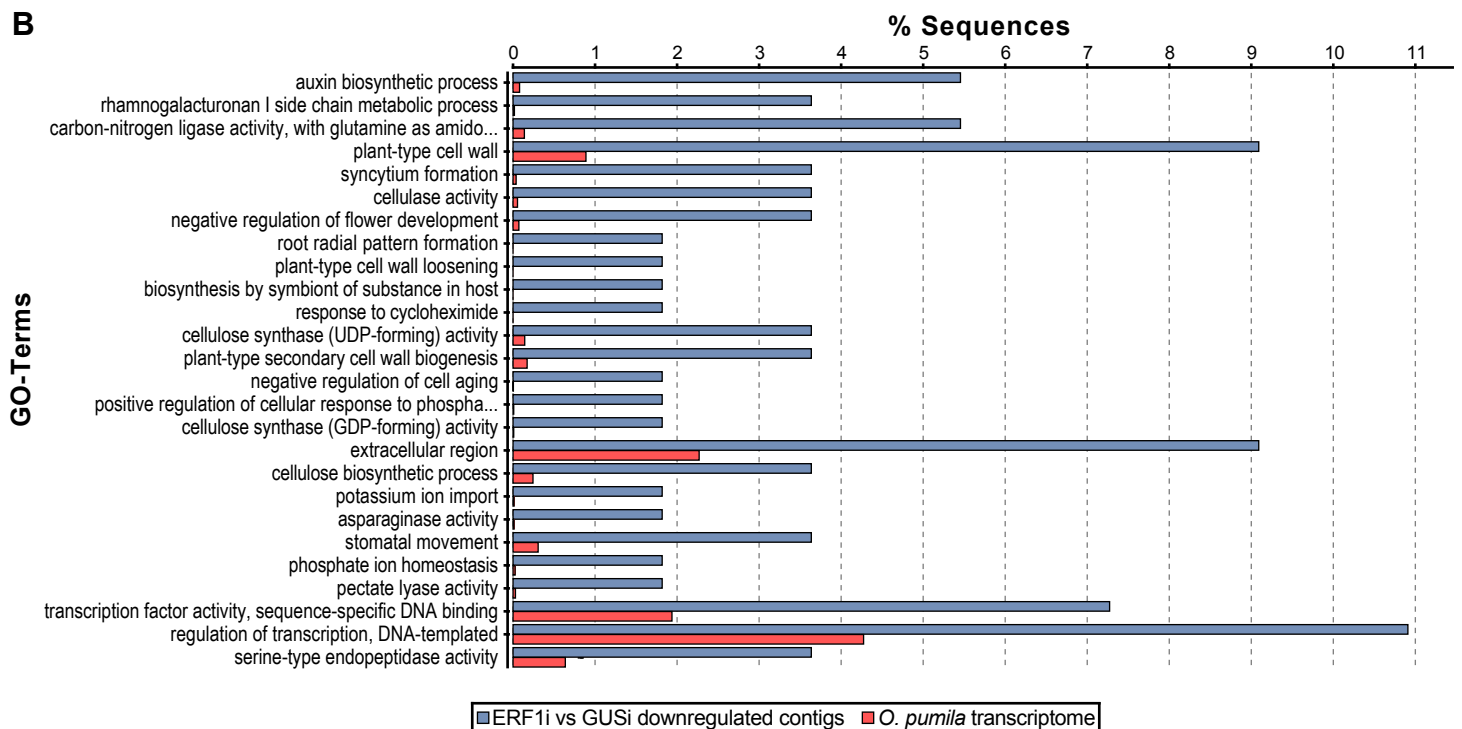

Supplement: Figure S7 — Gene ontology enrichment of differentially expressed transcripts in ERF1i. GO enrichment analysis of (A) upregulated and (B) downregulated transcripts in ERF1i compare to GUSi was performed using Fisher's exact test with a false discovery rate cut-off 0.05. Percentage of enriched GO terms statistically significant with respect to a reference set of O. pumila de novo transcriptome assembly are shown. [file Image7.PDF]

GUSi-9 GUSi-10 ERF1i-7 ERF1i-19 ERF2i-2 ERF2i-3

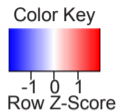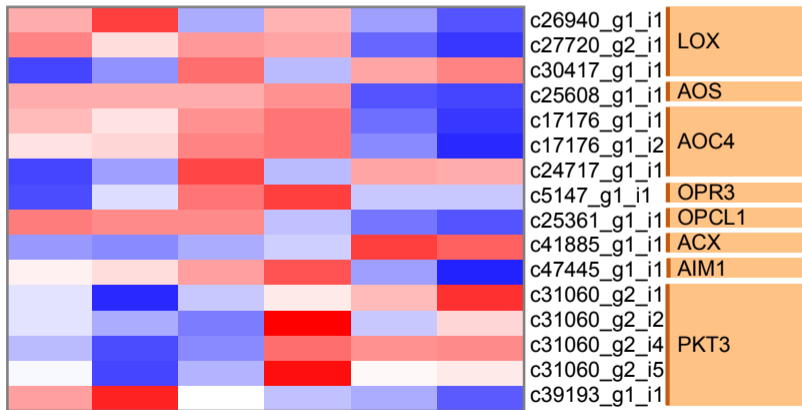

Supplement: Figure S8 — Heatmap diagram of expression levels of genes in jasmonic acid biosynthesis pathway. The heatmap was constructed using FPKM of the putative jasmonic acid biosynthetic genes. The expression levels are illustrated in blue-red scale. Blue indicates lower expression and red indicates higher expression. Enzyme genes and their corresponding contigs are listed on the right side. LOX, linoleate 13S-lipoxygenase; AOS, allene oxide synthase; AOC, allene oxide cyclase; OPR, 12-oxophytodienoate reductase; OPCL, OPC ligase; ACX, fatty acyl-CoA oxidase; AIM, enoyl-CoA hydratase/3-hydroxyacyl-CoA dehydrogenase; PKT, 3-keto-acyl-CoA-thiolase. [file Image8.PDF]
