# Supplementary material for: Function of AP2/ERF Transcription Factors Involved in the Regulation of Specialized Metabolism in Ophiorrhiza pumila Revealed by Transcriptomics and Metabolomics
Source: Front Plant Sci. 2016 Dec 9;7:1861. doi: 10.3389/fpls.2016.01861 (PMC5145908; doi:10.3389/fpls.2016.01861)
Supplement: Figure S2 — Amino acid sequence alignment of (A) OpERF1 to OpERF5, (B) OpERF1, and group VII Arabidopsis AP2/ERFs Op, Ophiorrhiza pumila; At, Arabidopsis thaliana. The sequences were aligned using ClustalW in Bioedit software version 7.2.5. AP2/ERF domain is enclosed in a black box. N-terminal MCGGAI(I/L) motif is enclosed in a red box. Asterisks indicate the reserved amino acids at position 14 and 19 of AP2/ERF domain. Solid bar indicates WLG motif. [file Image2.pdf]

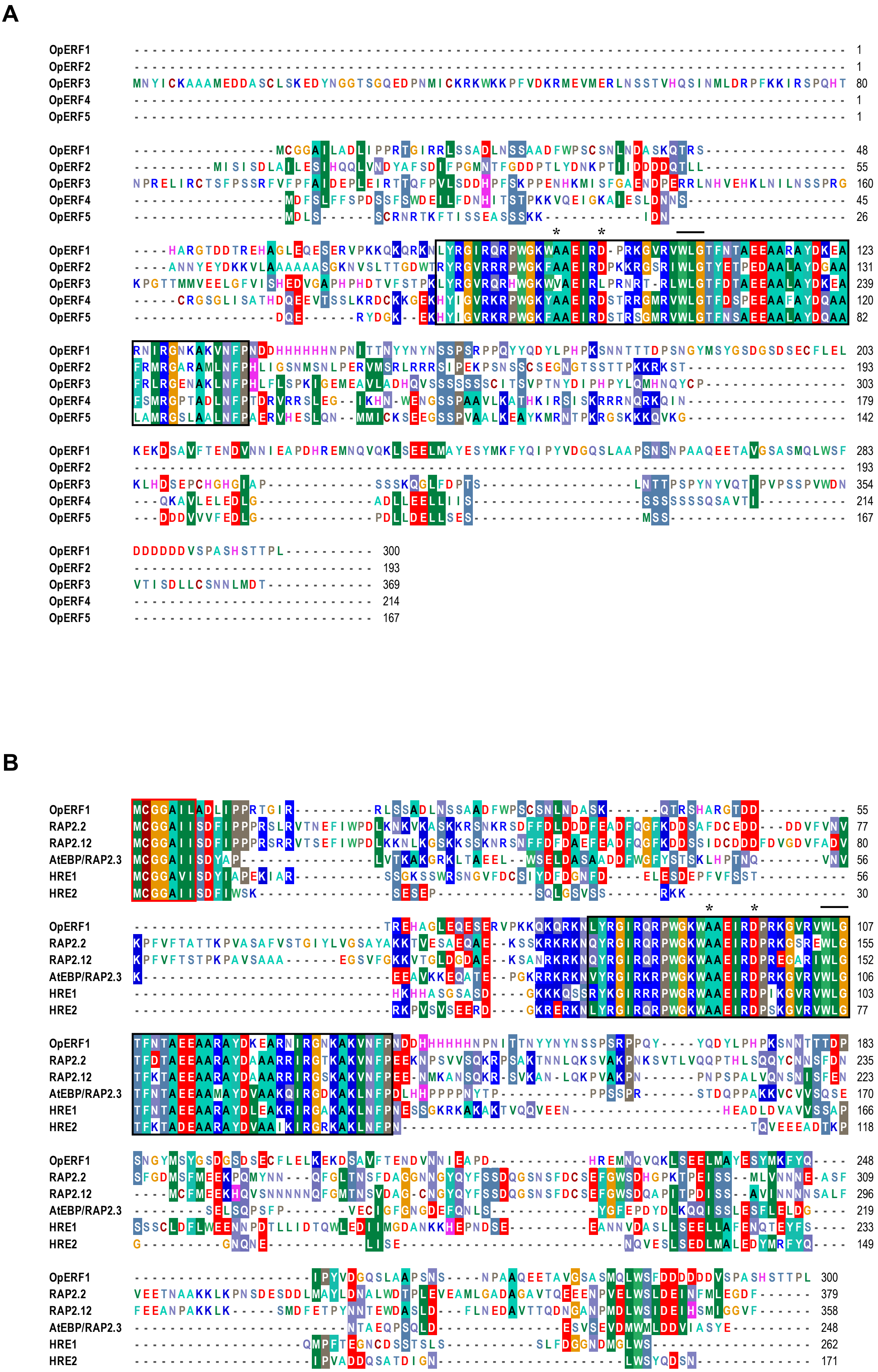
A
OpERF1
OpERF2
OpERF3
OpERF4
OpERF5
B
OpERF1
RAP2.2
RAP2.12
AtEBP/RAP2.3
HRE1
HRE2
