# Supplementary material for: Function of AP2/ERF Transcription Factors Involved in the Regulation of Specialized Metabolism in Ophiorrhiza pumila Revealed by Transcriptomics and Metabolomics
Source: Front Plant Sci. 2016 Dec 9;7:1861. doi: 10.3389/fpls.2016.01861 (PMC5145908; doi:10.3389/fpls.2016.01861)
Supplement: Table S1 — Primers used in qRT-PCR. [file Table1.DOCX]

**Supplementary Table S1** Primers used in qRT-PCR.

| **Gene** | **Primer type** | **Sequence（5'→3'）** |
| --- | --- | --- |
| *OpERF1* | Forward | CAAGAGGAGACAGCCGTAGG |
|  | Reverse | TACAGAGGCGTTGTGGAGTG |
| *OpERF2* | Forward | TCCTCACTTGATTGGCTCTAAC |
|  | Reverse | CTGGTATTGAACGCCTCCTC |
| *OpTDC* | Forward | GATTGGTCCCGATTTTCCTATG |
|  | Reverse | TCAATGGCAGCGGTTGAAG |
| *OpG10H* | Forward | TGGGATGGCCAAGGAAGTC |
|  | Reverse | GGAATTGATCGGCTGGAGAA |
| *OpSLS* | Forward | CTGGAGTTCTTCTCACTTGGACAA |
|  | Reverse | AGCACGATCTTGCCATTCG |
| *OpSTR* | Forward | ACCATCCTACGGACCAAACG |
|  | Reverse | CGACGGAAGCGTAAAGTTCAC |
| *OpTUB* | Forward | CCATTCCCACGACTCCACTT |
|  | Reverse | GAGCCCCTGGAGGTTAAAGG |
