# Supplementary material for: Function of AP2/ERF Transcription Factors Involved in the Regulation of Specialized Metabolism in Ophiorrhiza pumila Revealed by Transcriptomics and Metabolomics
Source: Front Plant Sci. 2016 Dec 9;7:1861. doi: 10.3389/fpls.2016.01861 (PMC5145908; doi:10.3389/fpls.2016.01861)
Supplement: Table S3 — Annotation and expression level of unigenes with AP2/ERF domain highly expressed in O. pumila hairy roots. [file Table3.DOCX]

**Supplementary Table 3** Annotation and expression level of unigenes with AP2/ERF domain highly expressed in *O. pumila* hairy root**s**.

| **Unigene** | **Length (bp)** | **Annotation** | **Accession number** | **RPKM** | |
| --- | --- | --- | --- | --- | --- |
|  |  |  |  | **CSC** | **HR** |
| Unigene36769_All | 1467 | RAP2-like protein [*Populus trichocarpa*] | ABQ62987.1 | 0 | 32.260 |
| Unigene20147_All | 348 | TINY-like protein [*Populus trichocarpa*] | ABQ62969.1 | 1.781 | 26.676 |
| **Unigene27166_All**  **(*OpERF1*)** | **633** | **ERF-like transcription factor [*Coffea canephora*]** | **AAS01337.1** | **0.587** | **21.229** |
| Unigene7684_All | 998 | TINY-like protein [*Populus trichocarpa*] | ABQ62969.1 | 1.056 | 19.385 |
| Unigene27079_All | 922 | AP2/ERF domain-containing transcription factor [*Populus trichocarpa*] | EEE95079.1 | 0.067 | 16.899 |
| Unigene3429_All | 1494 | AP2/ERF domain-containing transcription factor [*Populus trichocarpa*] | EEE88823.1 | 5.143 | 15.252 |
| Unigene27111_All | 1013 | AP2 domain-containing protein [*Oryza brachyanth*a] | BG73458.1 | 0 | 12.048 |
| Unigene25127_All | 1387 | AP2 domain-containing transcription factor [*Populus trichocarpa*] | EEF02630.1 | 0 | 11.374 |
| Unigene8856_All | 2020 | AP2/ERF domain-containing transcription factor [*Populus trichocarpa*] | EEE88823.1 | 4.816 | 10.156 |
| Unigene5195_All | 946 | AP2/ERF domain-containing transcription factor [*Populus trichocarpa*] | EEF02882.1 | 0.131 | 10.088 |
| Unigene27359_All | 621 | AP2 domain-containing transcription factor [*Populus trichocarpa*] | EEF03676.1 | 0.2 | 9.513 |
| Unigene31822_All | 1293 | AP2/ERF domain-containing transcription factor [*Populus trichocarpa*] | EEE84298.1 | 4.026 | 8.535 |
| Unigene11000_All | 526 | AP2 domain-containing transcription factor [*Populus trichocarpa*] | EEE82333.1 | 3.181 | 8.022 |
| Unigene15599_All | 1341 | AP2/ERF domain-containing transcription factor [*Populus trichocarpa*] | EEF00768.1 | 0 | 6.778 |
| **Unigene18425_All**  **(*OpERF2*)** | **666** | **ERF1 [*Nicotiana benthamiana*]** | **ADH04266.1** | **0.186** | **6.433** |
| **Unigene34283_All**  **(*OpERF4*)** | **683** | **ERF transcription factor 5 [*Nicotiana tabacum*]** | **AAU81956.1** | **0** | **6.368** |
| Unigene33203_All | 531 | AP2/ERF domain-containing transcription factor [*Populus trichocarpa*] | EEF00768.1 | 0 | 5.257 |
| Unigene61003_All | 1464 | RAP2-like protein [*Populus trichocarpa*] | ABQ62987.1 | 0 | 5.1 |
| Unigene60652_All | 401 | AP2/ERF domain-containing transcription factor [*Populus trichocarpa]* | EEE85331.1 | 0.309 | 4.695 |
| Unigene56827_All | 545 | AP2 domain-containing transcription factor [*Populus trichocarpa*] | EEF03676.1 | 0 | 4.526 |
| Unigene27059_All | 533 | AP2/ERF domain-containing transcription factor [*Populus trichocarpa*] | EEE99080.1 | 0 | 4.019 |
| Unigene33185_All | 1252 | AP2/ERF domain-containing transcription factor [*Populus trichocarpa*] | EEE82691.1 | 0 | 3.682 |
| Unigene19838_All | 436 | AP2/ERF domain-containing transcription factor [*Populus trichocarpa*] | EEE99080.1 | 0 | 3.127 |
| Unigene13014_All | 576 | AP2/ERF domain-containing transcription factor [*Populus trichocarpa*] | EEE82691.1 | 0.108 | 2.818 |
| Unigene5156_All | 518 | AP2 domain-containing transcription factor [*Populus trichocarpa*] | EEE78845.1 | 0 | 2.507 |
| Unigene12765_All | 431 | AP2 domain-containing transcription factor *[Populus trichocarpa*] | EEE77292.1 | 0 | 2.41 |
| Unigene12849_All | 299 | AP2 domain-containing transcription factor [*Populus trichocarpa*] | EEE88626.1 | 0 | 2.388 |
| Unigene4990_All | 392 | AP2 domain-containing transcription factor [*Populus trichocarpa*] | EEE88626.1 | 0 | 2.319 |
| **Unigene26293_All**  **(*OpERF3*)** | **322** | **AP2/ERF domain-containing transcription factor [*Populus trichocarpa*]** | **EEF07551.1** | **0** | **2.218** |
| **Unigene37445_All**  **(*OpERF5*)** | **679** | **ERF transcription factor 5 [*Nicotiana tabacum*]** | **AAU81956.1** | **0.365** | **2.199** |
| Unigene13658_All | 446 | AP2 domain-containing transcription factor [*Populus trichocarpa*] | EEF01440.1 | 0 | 1.747 |

Unigenes selected for RACE to obtain full-length are in bolded letter. bp, base pair; CSC, cell suspension culture; HR, hairy roots; RPKM, reads per kilobase of transcript per million mapped reads
